# Investigation of Molecular Iridium Fluorides IrF_*n*_ (*n*=1–6): A Combined Matrix‐Isolation and Quantum‐Chemical Study

**DOI:** 10.1002/chem.202104005

**Published:** 2022-03-18

**Authors:** Yan Lu, Yetsedaw A. Tsegaw, Artur Wodyński, Lin Li, Helmut Beckers, Martin Kaupp, Sebastian Riedel

**Affiliations:** ^1^ Freie Universität Berlin Institut für Chemie und Biochemie-Anorganische Chemie Fabeckstrasse 34/36 14195 Berlin Germany; ^2^ Technische Universität Berlin Institut für Chemie Theoretische Chemie/Quantenchemie Sekr. C7 Strasse des 17. Juni 135 10623 Berlin Germany

**Keywords:** IR spectroscopy, iridium fluorides, Jahn-Teller effect, laser-ablation, matrix-isolation, photochemistry, quantum-chemical calculations, spin-orbit effect, UV-vis spectroscopy

## Abstract

The photo‐initiated defluorination of iridium hexafluoride (IrF_6_) was investigated in neon and argon matrices at 6 K, and their photoproducts are characterized by IR and UV‐vis spectroscopies as well as quantum‐chemical calculations. The primary photoproducts obtained after irradiation with *λ*=365 nm are iridium pentafluoride (IrF_5_) and iridium trifluoride (IrF_3_), while longer irradiation of the same matrix with *λ*=278 nm produced iridium tetrafluoride (IrF_4_) and iridium difluoride (IrF_2_) by Ir−F bond cleavage or F_2_ elimination. In addition, IrF_5_ can be reversed to IrF_6_ by adding a F atom when exposed to blue‐light (*λ*=470 nm) irradiation. Laser irradiation (*λ*=266 nm) of IrF_4_ also generated IrF_6_, IrF_5_, IrF_3_ and IrF_2_. Alternatively, molecular binary iridium fluorides IrF_*n*_ (*n*=1–6) were produced by co‐deposition of laser‐ablated iridium atoms with elemental fluorine in excess neon and argon matrices under cryogenic conditions. Computational studies up to scalar relativistic CCSD(T)/triple‐*ζ* level and two‐component quasirelativistic DFT computations including spin‐orbit coupling effects supported the formation of these products and provided detailed insights into their molecular structures by their characteristic Ir−F stretching bands. Compared to the Jahn‐Teller effect, the influence of spin‐orbit coupling dominates in IrF_5_, leading to a triplet ground state with *C*_4v_ symmetry, which was spectroscopically detected in solid argon and neon matrices.

## Graphical abstract

**The IR spectra** of molecular iridium fluorides IrF_*n*_ (*n*=1–6) isolated in solid noble gas matrices are presented. Efficient formation of IrF_5_ together with minor IrF_3_ was achieved by irradiation of IrF_6_ with UV light, while subsequent irradiation leads to almost quantitative formation of IrF_4_. The presence of spin‐orbit coupling effects leading to a triplet ground state with *C*_4v_ symmetry of IrF_5_ was confirmed spectroscopically under cryogenic conditions.

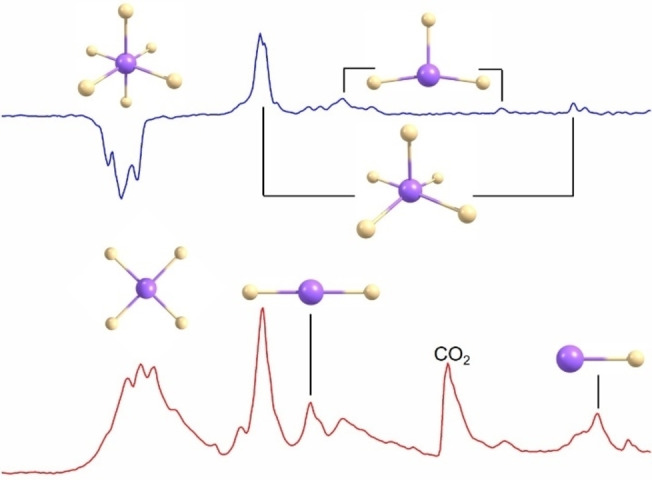

## Introduction

Iridium is one of the rarest transition metal elements in the earth‘s crust and its complexes have been efficiently utilized in catalytic water oxidation,^[1]^ C−H oxidation,^[2]^ biological probes^[3]^ and emitting materials.^[4]^ The most common oxidation states encountered for iridium‐complexes are +I and +III,^[5]^ but IrF_6_ is the most investigated and so far highest observed oxidation state of binary iridium fluoride species, and its synthesis dates back to 1929.^[6]^ Higher oxidation states +VIII in IrO_4_ ^[7]^ and +IX in [IrO_4_^+^]^[8]^ have recently been detected by infrared spectroscopy in the gas‐phase, and thus iridium has the widest range of oxidation states of any element, from ‐III to +IX.^[9]^ The higher oxidation states of binary iridium fluorides beyond +VI had not yet been confirmed experimentally. Computational studies predicted that IrF_7_ is a kinetically stabilized molecule and a good candiadate to be detected in the gas‐phase or matrix‐isolation studies, whereas IrF_8_ and IrF_9_ were shown to be metastable due to decomposition by strongly exothermic F_2_ elimination.^[10]^ Moreover, recent theoretical studies suggested that IrF_8_ can be stablized and obtained by the reaction of IrF_6_ and F_2_ under high pressure conditions.^[11]^

Numerous spectroscopic investigations of IrF_6_ in the gas‐phase and solid‐state are well documented.^[12]^ It was also shown that IrF_6_ has very similar crystallographic properties as the other molecular transition metal hexafluorides.^[13]^ However, experimental data on the molecular structure and spectroscopic studies of low‐valent iridium fluorides are missing, while some experimental and thermochemical studies have been reported.[^10^, ^14^] A systematic investigation that considers all possible iridium fluoride species is still lacking in the literature. IrF_5_ was studied in the gas‐phase by mass spectrometry as well as in the solid state by X‐ray diffraction, infrared and Raman spectroscopy, diffuse reflectance UV‐vis spectroscopy and magnetic susceptibility measurements.[^14b^, ^14c^, ^14d^] On the other hand, only the solid‐state data were reported for IrF_4_[^14c^, ^14f^, ^14g^, ^14h^] and IrF_3_.[^14i^, ^14j^] To the best of our knowledge, molecular IrF_2_ has not yet been studied spectroscopically, nor is its solid‐state structure known. Molecular IrF was observed and analyzed in *A* ^3^Φ_i_‐*X* ^3^Φ_i_ and *B* ^3^Φ_i_‐*X* ^3^Φ_i_ band systems only, using laser induced fluorescence and dispersed fluorescence spectroscopy.[^14k^, ^14l^]

Herein, we report a combined experimental and quantum‐chemical investigation of a series of molecular iridium fluorides IrF_*n*_ (*n*=1–6). Different methods were applied to produce these species under matrix‐isolation conditions at 6–12 K. First, it is well‐known that the matrix‐isolation infrared spectroscopic studies on the reaction products by co‐deposition of laser‐ablated transition metal atoms and fluorine is particularly useful for the generation of highly fluorinated species.^[15]^ Thus, the reaction between laser‐ablated iridium atoms and fluorine (0.5 and 1 %) in an excess of nobel gases (neon or argon) were carried out. Alternatively, binary iridium fluorides were generated by a photo‐initiated defluorination of IrF_6_ in solid neon and argon matrices under cryogenic conditions, allowing a systematic comparison of the results of both methods. The assignments of the obtained binary iridium fluoride species are further supported by quantum‐chemical calculations up to scalar relativistic coupled cluster CCSD(T)^[16]^ calculations and up to two‐component quasirelativistic DFT calculations including spin‐orbit coupling (SOC) effects.^[17]^

## Results and Discussion

### Computational results

**Figure 1:**
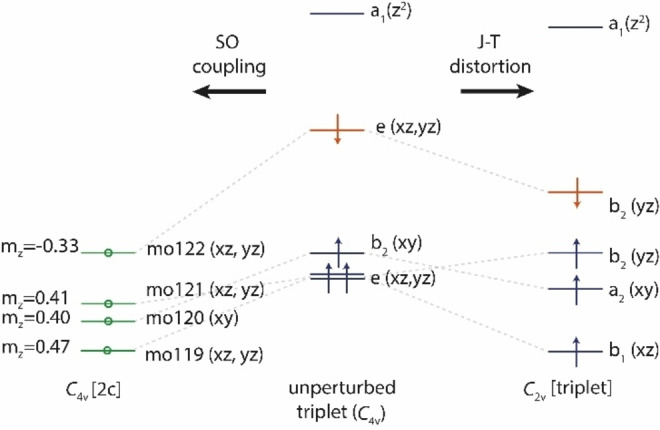
Simplified scheme of Jahn‐Teller (JT) distortion and spin‐orbit coupling (SOC) on the iridium 5d orbital splitting of triplet pyramidal IrF_5_. The energy levels for the unperturbed *C*_4v_ triplet state were modeled from an unrestricted calculation by spatial averaging of the b_1_ and b_2_ levels of a *C*_2v_‐symmetrical wave function to the e‐level that is shown. See Figure S1 for the difference energy values.

**Figure 2:**
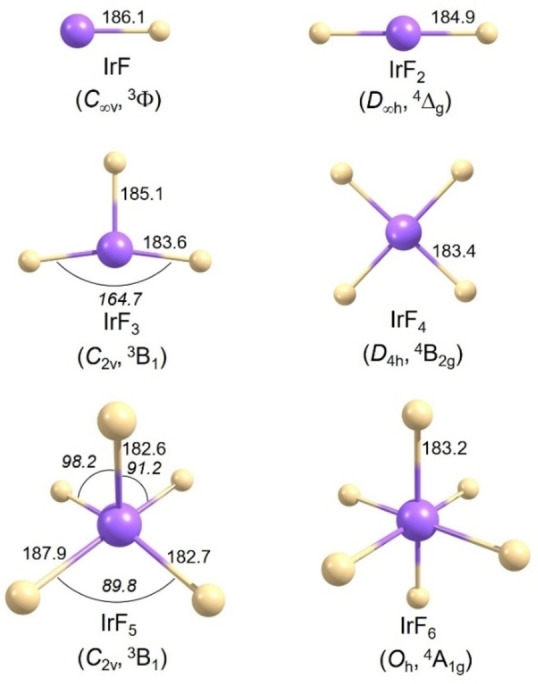
Computed structures of molecular iridium fluorides IrF_*n*_ (*n*=1–6) at the scalar‐relativistic pseudopotential CCSD(T) level. Selected bond lengths (pm) and angles (°, in *italics*) are shown. A complete list of the computed structures is given in Tables S1‐S5.

**Figure 3:**
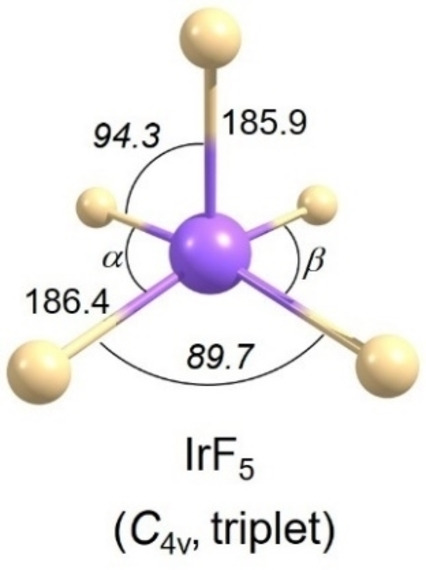
Computed structure of molecular IrF_5_ at the 2c‐X2C‐B3LYP/x2c‐TZVPall‐2c level. Selected bond lengths (pm) and angles (°, in *italics*) are shown. The computed structures of other iridium fluorides at this level are shown in Figure S3.

The electronic structure of the binary iridium fluorides IrF_*n*_ (*n*=1–7) and of the IrF_4_ ⋅ F_2_ complex were initially calculated at the DFT and CCSD(T) levels (using scalar relativistic pseudopotentials), considering all reasonable spin multiplicities. Subsequently, one‐ (1c‐X2C) and two‐component (2c‐X2C) all electron DFT calculations with the exact two‐component (X2C) Hamiltonian were carried out on IrF_n_ to evaluate the importance of SOC effects (see Figure 1). Optimized structures are shown in Figures 2, 3 and S3, and vibrational frequencies are compiled in Table 1 and Tables S5–S10 in Supporting Information. The bond length of diatomic IrF calculated by Kalamse and co‐workers at the MP2 level was 192.8 pm,^[14m]^ while our value of 186.1 pm at the CCSD(T) level is closer to the 185.1 pm obtained experimentally by laser induced fluorescence and dispersed fluorescence spectroscopy.^[14k]^ Siddiqui reported that IrF_2_ has a bent structure with Ir−F bond lengths of 189.7 pm at B3LYP DFT level with a scalar relativistic PP using unspecified basis sets.^[14n]^ However, according to our calculations, IrF_2_ is linear with a CCSD(T) bond length of 184.9 pm. Similar to the structure of AuF_3_ described in the literature,^[18]^ the triplet ground state of IrF_3_ also exhibits a planar T‐shaped structure with one long (185.1 pm) and two short (183.6 pm) Ir−F bond lengths. Of all possible structures for IrF_4_, a square‐planar structure (^4^B_2g_/*D*_4h_ symmetry) is the most stable with a bond length of 183.4 pm. The ^2^B_3g_/*D*_2h_ and ^2^B_2_/*D*_2d_ states for IrF_4_ are less stable than the ^4^B_2g_/*D*_4h_ ground state by up to 109.3 kJ mol^−1^ and 154.6 kJ mol^−1^, respectively (Table S1).

**Table 1:** Calculated and experimentally observed IR frequencies of Ir−F stretching modes of molecular iridium fluorides.^[a]^

Molecule	Mode	Calc. (Int.)			Exp.	
		CCSD(T)^[c]^	1c‐X2C‐B3LYP	2c‐X2C‐B3LYP	Ne	Ar
IrF^[b]^ (*C*_∞v_, ^3^Φ)	∑^+^	632.6	639 (100)	652 (100)	643.6	629.5
IrF_2_ (*D*_∞h_, ^4^Δ_g_)	∑_u_^+^	709.8	709 (176)	701 (158)	690.1	676.8
IrF_3_ (*C*_2v_, ^3^B_1_)	A_1_	710.6	700 (0)	680 (9)	–^[d]^	–^[d]^
	B_2_	708.3	699 (200)	689 (181)	684.9	–^[d]^
	A_1_	663.9	655 (62)	651 (42)	658.8	–^[d]^
IrF_4_ (*D*_4h_, ^4^B_2g_)	E_u_	727.9	716 (183)×2	715(161), 713 (168)	719.6/717.5/715.5	712.1
IrF_5_^[g]^ (*C*_4v_, triplet)				696 (1)	–^[d]^	–^[d]^
				690 (186)×2	701.3/697.8	689.0^[e]^, 685.9, 682.6^[e]^
				638 (37)	647.5/645.5	655.7
IrF_6_ (*O*_h_, ^4^A_1g_)	T_1u_		715 (100)×3	716 (100)×3	722.8/720.6/718.1	719.3/716.0^[f]^
IrF_7_ (*D*_5h_, ^3^A_1_′ )	A_2_“ E_1_′		718 (100) 662 (74)×2	718 (100) 663 (73) 661 (80)

[a] The complete set of calculated frequencies is provided in Supporting Information (Tables S5‐S10). Frequencies in cm^−1^ and intensities are shown in %. For the CCSD(T) calculations no intensities are available. [b] The frequency of IrF in the gas‐phase was reported at 650 cm^−1^.^[14k]^ [c] aug‐cc‐pVTZ‐PP basis sets. [d] Bands not observed, or too weak. [e] Matrix site. [f] Ref.^[12f]^. [g] Computed frequencies (in cm^−1^ ) of IrF_5_ (*C*_2v_, ^3^B_1_) obtained at the CCSD(T)/aug‐cc‐pVTZ‐PP level: 725.8, 724.4, 710.4, 682.5, 652.5 and at the 1c‐X2C‐B3LYP (relative intensities in parentheses): 720 (16), 706 (177), 695 (15), 667 (190), 637 (0).

Previously, it was reported that a square‐pyramidal quintet ground state of IrF_5_, with ^5^B_1_/*C*_4v_ symmetry is more favorable at scalar relativistic B3LYP/aT‐PP level than a ^3^A_1_/*C*_4v_ state which was computed to be slightly higher in energy by 19.7 kJ mol^−1^.^[14e]^ The e‐type orbital in the undistorted triplet *C*_4v_ state of IrF_5_ (Figure 1) breaks spin‐symmetry and orbital degeneracy has to be lifted either by J‐T distortion or by SOC (the ^5^B_1_/*C*_4v_ state is a result of *C*_4v_ symmetry being imposed at scalar‐relativistic level). This assumption agrees with our computations. In scalar relativistic calculations at the CCSD(T)/aug‐cc‐pVTZ‐PP (or 1c‐X2C‐B3LYP all‐electron) level, IrF_5_ distorts to a ^3^B_1_/*C*_2v_ ground‐state structure with an axial Ir−F bond of 182.6 (183.5) pm and two equatorial long bonds of 187.9 (189.2) pm and two shorter bonds of 182.7 (184.1) pm (Figure 2 and Table S2), consistent with previously reported optimized structures.^[10]^ On the other hand, the inclusion of SOC effects at 2c‐X2C‐B3LYP level (Figure 3) leads to a square‐pyramidal structure (triplet state, *C*_4v_ symmetry) for which a geometric parameter *τ*=0 was determined.^[19]^ Following the procedures described by Addison et al. with *τ*=(*β*‐*α*)/60° (*τ*=0, square pyramidal geometry; *τ*=1, trigonal bipyramidal geometry), where *β* and *α* (Figure 3) are the largest angles in the coordination sphere, *τ*=0 (*β*=*α*=89.7°) stands for perfectly square pyramidal geometries (*C*_4v_). For more angle details, see Table S2 in the Supporting Information. The 2c‐X2C‐B3LYP structure of IrF_5_ has a short axial Ir−F bond length of 185.9 pm and four longer bonds of 186.4 pm (Figure 3). That is, the computations suggest that SOC quenches the J‐T distortion observed for the triplet state at scalar relativistic levels. Upon introduction of SOC, a clear assignment of a molecular term symbol becomes more difficult, as spin ceases to be a good quantum number. We nevertheless assign the ground state to be a *C*_4v_ triplet state based on the length of its spin magnetization vector indicating two unpaired electrons (see values of m_z_ in Figure 1).

The calculated bond length of 183.2 pm for the quartet IrF_6_ ground state with its well‐known *O*_h_ structure shows good agreement with the gas‐phase electron diffraction value of 183.9 pm^[12a]^ as well as with the EXAFS value of 182.2 pm measured in the solid‐state.^[13]^ Furthermore, a pentagonal‐bipyramidal triplet ground‐state (*D*_5h_) for IrF_7_ has been reported previously^[10]^ at the B3LYP level of theory, which is consistent with our calculations (Figure S2).

## Experimental Results

### A. Photodecomposition of IrF_6_

**Figure 4:**
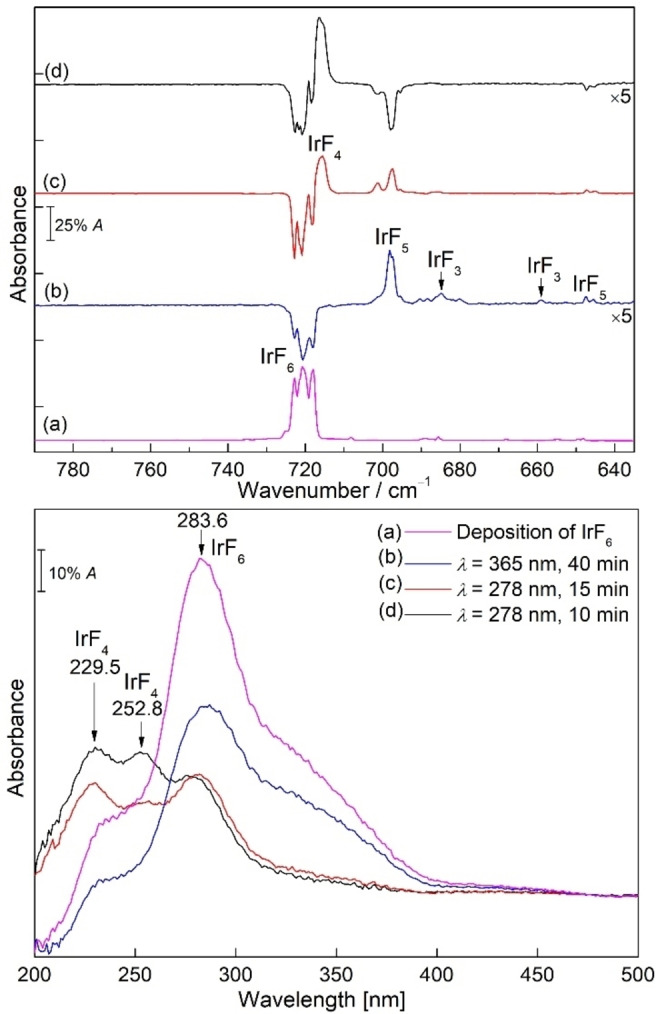
IR (top) and UV‐vis (bottom) spectra recorded from the same neon matrix at 6 K showing the photochemistry of IrF_6_. (a) Spectra of IrF_6_ obtained after deposition for 30 min (pink lines), (b) UV‐vis and difference IR spectra obtained after *λ*=365 nm irradiation for 40 min (blue lines), (c) subsequent irradiation of the same matrix at *λ*=278 nm for 15 min (red lines), and (d) subsequent irradiation of the same matrix at *λ*=278 nm for 10 min (black lines). Upward bands in the difference spectra are formed at the expense of downward bands.

**Figure 5:**
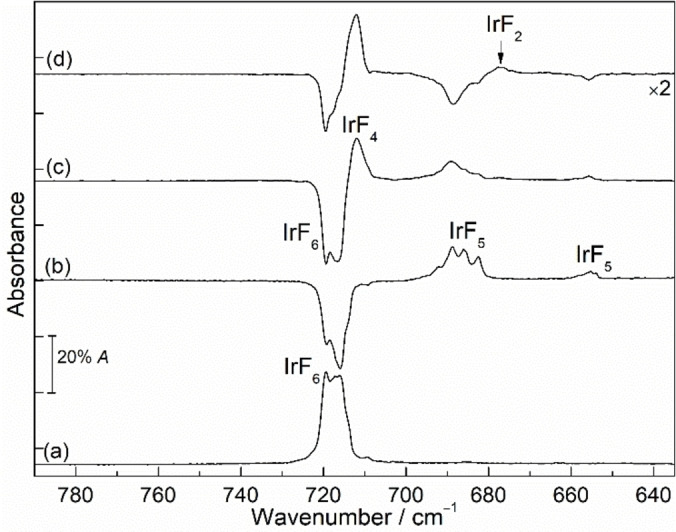
IR spectra in argon matrix at 6 K showing the photochemistry of IrF_6_. (a) Spectrum of IrF_6_ obtained after deposition for 40 min, (b) difference IR spectrum obtained after *λ*=365 nm irradiation for 60 min, (c) subsequent irradiation of the same matrix at *λ*=278 nm for 15 min, and (d) subsequent irradiation of the same matrix at *λ*=278 nm for 30 min. Upward bands in the difference spectra are formed at the expense of downward bands.

Figure 4 reveals the IR and UV‐vis spectra of iridium hexafluoride before and after irradiation (*λ*=365 and 278 nm) in neon matrix at 6 K. Similar experiments in argon matrix are also shown in Figure 5 and additional results are provided in Supporting Information (Figures S5–S10). A comparison between the experimentally observed and calculated frequencies is shown in Table 1.

#### UV‐vis experiments

To understand the photochemical decomposition behavior of IrF_6_ in detail, the IR and UV‐vis spectra of IrF_6_ in the same neon matrix under cryogenic conditions at 6 K are shown in Figure 4. These experiments allow to correlate changes in band intensities in the IR and UV‐vis regions and confirm the band assignments of the newly formed binary iridium fluoride species. We will first discuss the UV‐vis results, and the IR results will be described in the next sections.

The UV‐vis spectrum of IrF_6_ in neon matrix at 6 K shows continuous absorption in this region (200–500 nm). The absorbance contains mainly the intense broad bands at 283.6 and 241.8 nm and a distinct, relatively intense shoulder at 336.8 nm, which is in good agreement with the reported values in nitrogen matrix.^[12f]^ Detailed UV studies of IrF_6_ including vibrational progression and transitions have been discussed in the literature.^[12f]^ After 40 min of UV light photolysis (*λ*=365 nm), the intensity of the absorption bands at 241.8, 283.6, and 336.8 nm decreased by half, indicating the photo‐initiated defluorination of IrF_6_ to the lower‐valent iridium fluorides. However, no obvious new absorption UV bands have been identified to allow a further assignment of possible decomposition products. Robinson and Westland described earlier that IrF_4_ is formed by irradiation of IrF_6_ with UV light.^[14a]^ But later Bartlett and Rao corrected the previous assignment to IrF_5_,^[14b]^ suggesting the photodecomposition of IrF_6_ to the lower‐valent iridium fluorides.

Previously the visible spectra of IrF_5_ in HF solution were reported with two absorption bands at 683 and 840 nm.^[14c]^ Later, the diffuse reflectance UV‐vis spectrum for solid IrF_5_ with strong absorption bands in a broad range from about 200 to 500 nm, 840, and 1524 nm and similarly for IrF_3_ at 256, 300, and 500 nm were published.[^14d^, ^14j^] Additionally, the electronic diffuse reflectance spectrum of solid IrF_4_ was characterized at 263, 320, 405 and 510 nm.^[14h]^ To the best of our knowledge no UV‐vis studies for IrF_2_ and IrF have been reported so far. However, our attempts to obtain a clear UV absorption band for molecular IrF_5_ were so far unsuccessful, probably because of the overlapping absorption of IrF_6_ and the very low abundance of the molecule and the detection range of our UV‐vis spectrometer (200–850 nm).

In addition, subsequent irradiation of the same matrix at *λ*=278 nm for 15 min produced two distinct new bands at 229.5 and 252.8 nm in the UV‐vis spectrum, while the absorption band at 283.6 nm of IrF_6_ was slightly reduced (Figure 4c, bottom trace). Further irradiation at the same wavelength increased the newly formed bands at 229.5 and 252.8 nm, which could be tentatively assigned to IrF_4_ based on IR data (Figure 4d). However, the difference in the rate of rise of the two bands upon further 10 min irradiation at *λ*=278 nm suggests that the assigned absorption band of IrF_4_ at 229.5 nm may have overlapped with the band of other binary iridium fluorides.

#### IR experiments

In analogy to the UV‐vis experiments, several IR experiments were performed to understand the photochemistry of IrF_6_, and the species produced by irradiation. The IR band positions of iridium hexafluoride in argon and nitrogen matrices were previously reported by Holloway^[12f]^ and agree very well with our experimental results obtained in neon and argon matrices (Figures 4, 5 and S5–S10). In the neon matrix, the bands of IrF_6_ split into three components with almost equal intensity at 722.8, 720.6 and 718.1 cm^−1^ whereas in argon only two bands at 719.3 and 716.0 cm^−1^ are observed, explained by the weak interactions with the atoms in the matrix host.

Irradiation with UV light (*λ*=365 nm) of IrF_6_ isolated in a neon matrix resulted in the decline of all IR absorptions of IrF_6_ and formation of a complex spectrum, with the strongest IR bands at 697.8 cm^−1^ and other weaker bands at 684.9, 658.8, 647.5 and 645.5 cm^−1^ (Figure 4b, top trace). In order to distinguish these IR bands, the matrix was further irradiated at 278 nm for 15 min. The corresponding IR difference spectrum (Figure 4c, top trace) demonstrates an increase of the aforementioned three IR bands at 697.8, 647.5 and 645.5 cm^−1^ as well as a new IR band at 717.5 cm^−1^ and concurrently a decrease of bands of IrF_6_ at 722.8, 720.6 and 718.1 cm^−1^. Interestingly, attempts to increase the abundance of the newly produced species by prolonged irradiation with 278 nm resulted in an intensity decrease of the bands at 697.8, 647.5 and 645.5 cm^−1^, while the intensity of the IR band at 717.5 cm^−1^ increased significantly (Figure 4d, top trace). The prololonged irradiation (*λ*=278 nm) completely destroyed the IR bands of IrF_6_ and clearly indicated that the band at 717.5 cm^−1^ belongs to a new species whose absorption is close to that of the precursor IrF_6_ (Figures S5 and S6).

In a separate analogy experiment, the neon matrix containing the 365 nm photolysis product of IrF_6_ was irradiated with a blue‐light source (*λ*=470 nm) for 15 minutes (Figure S7). This resulted in a decrease of the IR bands at 697.8, 647.5, and 645.5 cm^−1^ and an increase of the IR bands of IrF_6_. Concomitantly, a new band at 717.5 cm^−1^ and two weak bands at 684.9 and 658.8 cm^−1^ are formed (Figure S7).

Similarly, the UV‐light (*λ*=365 nm) irradiation of IrF_6_ was also performed in solid argon matrix (Figure 5). This irradiation produced a broad band centered at 685.9 cm^−1^ and a weak band at 655.7 cm^−1^, which are comparable to the observation of a sharp band at 697.8 cm^−1^ and other weak bands in the neon matrix experiments discussed above. Furthermore, both sets of bands show similar photochemical behavior in the subsequent 278 nm or 470 nm irradiations (Figures 5c, 5d and S8). A strong band at 712.1 cm^−1^ that is very close to the band of IrF_6_ was also observed, and a weak band at 676.8 cm^−1^ becomes apparent upon prolonged irradiation at 278 nm (Figure S6).

Based on the changes in the IR spectra, obtained at different photolysis wavelengths and times, and annealing behavior, in comparison with UV‐vis data obtained in the same matrix experiments, and using further support by quantum‐chemical calculations, we were able to assign the newly formed species to the low‐valent iridium fluorides IrF_*n*_ (*n*=1–5). The IR bands at 697.8, 647.5 and 645.5 cm^−1^ in solid neon (685.9 cm^−1^ and 655.7 cm^−1^ in solid argon) produced in the photolysis of IrF_6_ can be grouped and belong to different vibrational modes of the same new molecule. For the photolysis products of IrF_6_, the most likely candidates are IrF_5_ and IrF_4_, which could be produced by homolytic Ir−F bond cleavage and by elimination of F_2_. Quantum‐chemical calculations were performed to support the assignments, and the calculated IR spectra of the binary iridium fluorides are summarized in Table 1 as well as in Tables S1–S11. Craciun and co‐workers reported that the calculated frequencies of IrF_5_ in the ^5^B_1_/*C*_4v_ ground state are 702 and 526 cm^−1^ with an intensity distribution of about 7 : 1.^[14e]^ In addition, IrF_5_ with a *C*_2v_ triplet ground state was also mentioned,^[10]^ for which two different Ir−F stretching bands with almost identical intensities would be expected (Table S7). However, these predicted positions and intensities of the bands do not agree well with our experimental values. This may be explained by the influence of SOC for the IrF_5_ system. Inclusion of SOC for the triplet state of IrF_5_ with *C*_4v_ structure at the 2c‐X2C‐B3LYP level gave harmonic IR frequencies at 690 and 638 cm^−1^, which are in good agreement with the observed band positions at 697.8, 647.5 and 645.5 cm^−1^ in neon and at 685.9 and 655.7 cm^−1^ in argon matrices, respectively (Tables 1 and S7). The bands at 689.0 and 682.6 cm^−1^ in argon were assigned to the matrix site bands of IrF_5_ based on the behavior during annealing, where these bands quickly disappeared (Figure S9).

Moreover, the two very weak bands at 684.9 and 658.8 cm^−1^ observed after 365 nm irradiation of IrF_6_ were assigned to IrF_3_, in agreement with the calculations for the T‐shaped planar structure of IrF_3_ at B3LYP and CCSD(T) levels (Table 1). This species is likely formed upon further photolysis of IrF_5_ generated in the matrix experiments. However, the absorption of IrF_3_ identified in neon could not be detected in the argon matrix, probably due to its low abundance or overlap with the broad IR bands of IrF_5_. According to the calculated vibrational displacement vectors, the bands at 684.9 cm^−1^ and 658.8 cm^−1^ correspond to the asymmetric stretching vibrations of F−Ir−F and the stretching vibrations of Ir−F, which both have large blue‐shifts of 33.3 cm^−1^ and 96.7 cm^−1^, respectively, compared to the stretching vibrations in NIrF_3_ (651.6 and 562.1 cm^−1^, Ne‐matrix).^[20]^ This is in agreement with the absence of a frequency for the remaining F−Ir−F symmetric stretching frequency of IrF_3_ in the recorded spectra, for which a very low intensity was predicted (Table 1).

Next, bands for the new IrF_4_ molecule are observed at 717.5 cm^−1^ in Ne and 712.1 cm^−1^ in Ar matrices under cryogenic conditions at 6 K. As shown in Figures S5 and S6, the formation of IrF_4_ by 278 nm irradiation of IrF_6_ is almost quantitative in both solid neon and argon matrices. The assignment to this molecule is based on the basis of our experimental observations. For a tetrahedral structure, only a single infrared active absorption would be expected in the Ir−F stretching vibration region, as recently discussed in detail for the analogous PtF_4_ molecule.^[21]^ Formation of IrF_5_ and IrF_4_ is evident after short irradiation of IrF_6_ with 278 nm. However, the efficient formation of IrF_4_ and the depletion of the initially generated IrF_5_ under prolonged 278 nm irradiation indicate the formation of IrF_4_ as photolysis product of IrF_5_ instead of as a product of F_2_‐elimination from IrF_6_. The CCSD(T) calculations predict a square‐planar structure for molecular IrF_4_, and the observed frequencies are consistent with the calculated fundamental IR frequencies at 727.9 cm^−1^ (Table 1). It is noteworthy that solid‐state IR data of IrF_4_ showed a strong iridium‐fluorine bridging stretching vibration located at around 550 cm^−1^.^[14c]^ As expected, no corresponding IR band for IrF_4_ has been detected in the range of 500–600 cm^−1^ in our experiments (Figures S5 and S6). Furthermore, laser irradiation (*λ*=266 nm) of IrF_4_ produced IR bands of IrF_6_, IrF_5_, IrF_3_ and an unknown new band at 690.1 cm^−1^ in the neon matrix (Figure S10).

Similarly, the argon matrix containing the 365 nm photolysis products of IrF_6_ was further subjected to 278 nm irradiation (Figure 5d). In addition to the dominant formation of the IR band of IrF_4_ at 712.1 cm^−1^, a new carrier also appeared after photolysis (278 nm) with a weak but distinguishable IR band at 676.8 cm^−1^, which continued to grow slightly with longer photolysis. This carrier could be a photolysis product from decomposition of IrF_4_. Recall that the new band at 690.1 cm^−1^ was produced upon laser irradiation (*λ*=266 nm) of IrF_4_ in neon matrix. Similar to the observed shifts of neon to argon matrix of PtF_2_ (Δν=−14.5 cm^−1^),^[21]^ the assignment of the new band at 690.1 cm^−1^ in neon (and 676.8 cm^−1^ in argon) to IrF_2_ in the current experiment is plausible when assuming a reasonable blue shift in the neon matrix (shift to argon: Δν=−13.2 cm^−1^). Also, the observed band positions are consistent with the strongest fundamental IR vibration at 709.8 cm^−1^ calculated at the CCSD(T) level for the linear IrF_2_ molecule (Table 1) and associated with the asymmetric F−Ir−F stretching vibration.

### B. Reaction of laser‐ablated iridium atoms with fluorine

**Figure 6:**
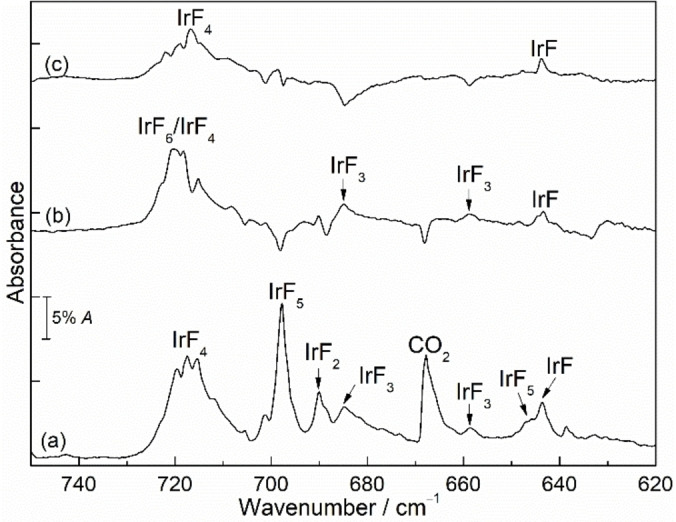
IR spectra in neon matrix at 6 K. (a) IR spectrum of reaction products of laser‐ablated Ir atoms with 1 % F_2_. Difference IR spectrum obtained after (b) *λ*=470 nm irradiation for 20 min and (c) subsequent *λ*=278 nm irradiation for 25 min.

Alternatively, the binary iridium fluorides were synthesized by the reaction of laser‐ablated iridium atoms with fluorine diluted in excess neon and argon under cryo‐conditions at 6 K. This method has also been used successfully for the synthesis of other metal fluorides in our group.[^15^, ^21^] Figure 6 shows spectra obtained after deposition of laser‐ablated iridium atoms with 1 % fluorine in solid neon followed by irradiation at different wavelengths. This experiment supported our assignments obtained from the photolysis of IrF_6_: five groups of absorptions were observed, four of which above have been assigned to IrF_5_, IrF_4_, IrF_3_ and IrF_2_, whereas an additional band observed at 643.6 cm^−1^ was unknown before. Irradiation of the same neon matrix by the blue‐light (470 nm) leads to a decrease in the IrF_5_ bands and an increase in the corresponding IrF_6_, IrF_4_, and IrF_3_ bands, while the band at 643.6 cm^−1^ increases slightly, but there is no noticeable change in the IrF_2_ band (Figure 6b). This means that the new band at 643.6 cm^−1^ belongs to a new species not observed in our experiments before. Subsequent irradiation at 278 nm further destroyed the bands of IrF_3_ and increased the intensity of IrF_4_ as well as the unassigned band at 643.6 cm^−1^.

**Figure 7:**
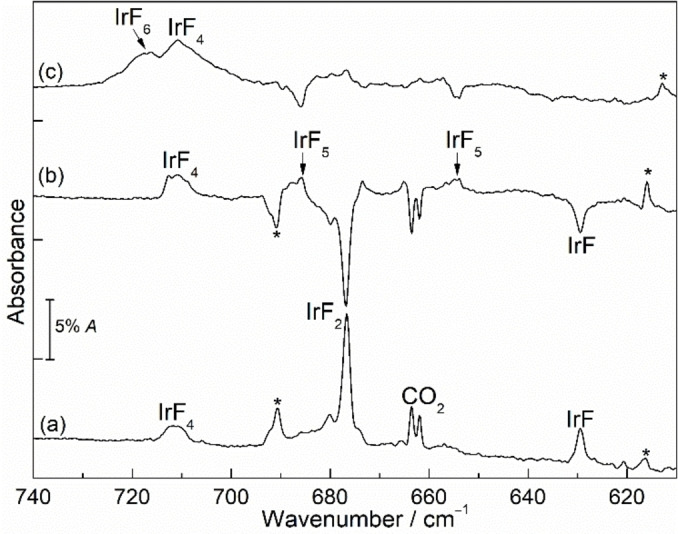
IR spectra in argon matrix at 12 K. (a) IR spectrum of reaction products of laser‐ablated Ir atoms with 0.5 % F_2_. (b) Difference IR spectrum obtained after annealing to 35 K and (c) subsequent *λ*=470 nm irradiation for 30 min. The bands marked with asterisks are assigned to unknown impurities.

Analogous spectra were recorded after sample deposition in argon (Figure 7). There are two remarkable differences between the neon and argon experiments. One is that a strong, sharp band of IrF_2_ in argon corresponds to a weaker absorption in the neon matrix. The other is that IrF_5_ is the major product after sample deposition when neon was used as the matrix host, whereas the corresponding bands in the argon matrix only appeared after annealing to 35 K. Surprisingly, the spectra show no evidence for the formation of a trifluoride in argon. New bands at 690.8 and 629.5 cm^−1^ that were also observed in argon on deposition, decreased substantially during annealing of the sample. The higher band at 690.8 cm^−1^ is nearly unaffected by photolysis. It could not be assigned properly, although we considered the formation of dimers and charged species.

The lower observed bands mentioned above at 643.6 cm^−1^ in neon and at 629.5 cm^−1^ in argon after deposition could be assigned as the Ir−F stretching vibrational mode of the diatomic IrF molecule (Figures 6 and 7). This assignment is in accordance with the fundamental gas‐phase frequency of IrF at 650 cm^−1^ deduced from electronic band spacings.^[14k]^ Our CCSD(T) calculations predict a strong stretching frequency for this species at 632.6 cm^−1^, slightly lower than the above values obtained in neon matrix (Table 1), but significantly blue‐shifted in comparison to the reported value (575.0 cm^−1^) obtained previously at second‐order Moller‐Plesset (MP2) perturbation theory level.^[14m]^

The theoretically predicted structure of *D*_5h_ symmetry of IrF_7_ would have two bands at 718 and 662 cm^−1^ with an intensity distribution of about 3 : 4 (Table 1). Unfortunately, all attempts to detect IR bands of a higher iridium fluoride such as IrF_7_, that has been regarded as a candidate species in matrix‐isolation studies,^[10]^ by additional photolysis of the deposits were not successful. In addition, similar to our findings on the interaction between PtF_4_ and F_2_ in the matrix,^[21]^ the possible formation of difluorine complexes IrF_4_ ⋅ F_2_ and IrF_5_ ⋅ F_2_ based on fluorine‐specific interactions between the metal fluoride and elemental F_2_ was investigated. The optimized structures of IrF_4_ ⋅ F_2_ complex were obtained with both side‐on and end‐on coordination of fluorine to iridium at the B3LYP‐D3/aug‐cc‐pVTZ‐PP level (Figure S4), and the energy difference between these two structures is relatively small (1.7 kJ mol^−1^). The optimization of the IrF_5_ ⋅ F_2_ complex failed due to initiation of F−F bond cleavage at the B3LYP‐D3/aug‐cc‐pVTZ‐PP level, indicating the already strong Lewis character of the IrF_5_ species that leads to the formation of IrF_6_ and a F atom. Furthermore, the predicted Ir−F stretching frequency of the IrF_4_ ⋅ F_2_ complex is only 1.5 cm^−1^ higher than that for isolated IrF_4_ (B3LYP‐D3/aug‐cc‐pVTZ‐PP level; Table S11). Therefore, the presence of the IrF_4_ ⋅ F_2_ complex in our spectra could not be completely excluded, as it could overlap with the strong band of isolated IrF_4_.

#### Computed thermochemical data

Having already assigned the experimentally obtained iridium fluorides, we now propose the possible decomposition channels and computationally analyze the thermochemical stability of the observed compounds (Table 2). This technique has also been used previously in the literature to examine the stability of molecular fluorides under cryogenic conditions.[^15a^, ^22^] Based on our experimental observations in argon and neon matrices, concerted elimination of F_2_ and homolytic cleavage of one iridium‐fluorine bond were considered for IrF_*n*_ (*n*=1–6). These decomposition channels of IrF_6_ are strongly endothermic at the 2c‐X2C‐B3LYP and CCSD(T) levels (Table 2) as well as at B3LYP/aT‐PP and 1c‐X2C‐B3LYP levels (Table S12). Therefore, it should be possible to obtain the IrF_5_ and IrF_4_ molecules under appropriate conditions, for example using the matrix isolation techniques shown in this work. Similarly, the value of 204.0 kJ mol^−1^ at 2c‐X2C‐B3LYP level obtained for the IrF_5_→IrF_4_+F reaction indicates a low thermal stability of molecular IrF_5_, which is consistent with our experimental results that the initially generated IrF_5_ can be transformed into other binary fluorides upon 470 or 278 nm irradiations. The calculated thermochemistry predicts the low‐valent fluorides IrF and IrF_2_ to be stable against fluorine elimination and homolytic bond cleavage.

**Table 2:** Computed thermochemical stability of iridium fluorides (298.15 K, kJ mol^−1^) at different levels of theory.

Reaction	2c‐X2C‐B3LYP^[a]^		CCSD(T)^[b]^	
	Δ*E*+ΔZPE	Δ_r_ *H*	Δ*E*+ΔZPE(B3LYP)^[c]^	Δ_r_ *H* ^[d]^
IrF_6_→IrF_4_+F_2_	312.7	316.6	308.9	312.5
IrF_6_→IrF_5_+F	256.0	260.5	310.1	315.4
IrF_5_→IrF_3_+F_2_	421.3	424.6	374.4	376.5
IrF_5_→IrF_4_+F	204.0	207.0	145.0	146.9
IrF_4_→IrF_2_+F_2_	507.2	510.6	526.2	529.4
IrF_4_→IrF_3_+F	364.6	368.5	375.7	379.4
IrF_3_→IrF+F_2_	607.2	609.5	654.1	656.2
IrF_3_→IrF_2_+F	289.9	293.1	296.8	299.8
IrF_2_→Ir+F_2_	681.7	684.3	716.9	719.2
IrF_2_→IrF+F	464.5	467.3	503.6	506.2
IrF→ Ir+F	364.5	367.9	359.6	362.9

[a] x2c‐TZVPall‐2c basis sets. [b] aug‐cc‐pVTZ‐PP basis sets. [c] Using B3LYP zero point energy corrections for the electronic energies at CCSD(T) level. [d] The enthalpies at CCSD(T) level were calculated by adding the enthalpy corrections (B3LYP) to electronic energy changes.

## Conclusions

A series of molecular iridium fluorides IrF_*n*_ (*n*=1–6) were prepared by the reaction of laser‐ablated iridium atoms with elemental fluorine and by the photo‐initiated defluorination of IrF_6_, isolated in solid noble‐gas matrices. These fluorides were spectroscopically identified and supported by quantum‐chemical calculations. The species IrF_*n*_ (*n*=1–5) were produced for the first time under the cryogenic conditions, and their formation in the laser ablation experiments depends on the noble gas host, similar to the chemistry of platinum fluorides.^[21]^ However, efficient formation of IrF_5_ together with minor IrF_3_ products was achieved by irradiation into the absorption maxima of IrF_6_ in the UV region (*λ*=365 nm), while subsequent irradiation (*λ*=278 nm) leads to almost quantitative formation of IrF_4_ in both neon and argon matrices. Further irradiation into the absorption maxima of IrF_4_ (*λ*=266 nm) leads to the formation of IrF_6_, IrF_5_, IrF_3_ and IrF_2_ in neon matrices by addition and elimination of fluorine radicals and/or molecular fluorine. The assignment of these species was computationally supported by one‐ and two‐component quasirelativistic DFT methods and scalar‐relativistic CCSD(T) calculations. IrF_5_ is one of the very rare examples in which a significant influence of SO coupling on the structure is found, where a high‐symmetry (*C*_4v_) triplet structure is favored energetically at 2c‐X2C level over the Jahn‐Teller distorted ^3^B_1_/*C*_2v_ structure obtained at scalar relativistic levels. The presence of SOC effects leading to a triplet ground state with *C*_4v_ symmetry of IrF_5_ in solid neon and argon matrices was confirmed by the observed IR frequencies. Attempts to detect IR bands of higher iridium fluorides such as IrF_7_ and/or difluorine complexes of IrF_5_ or IrF_4_ were unsuccessful which is perhaps due to low yield or overlap of the bands.

## Experimental and Computational Details

The technique of matrix‐isolation infrared (IR) spectroscopy and laser‐ablation apparatus have been described in detail in previous works.[^15^, ^21^] Matrix samples were prepared by co‐deposition of laser‐ablated iridium atoms with 0.5 % and 1 % elemental fluorine diluted in neon (99.999 %, Air Liquide) or argon (99.999 %, Sauerstoffwerk Friedrichshafen). The stainless‐steel F_2_ storage cylinder was cooled in liquid nitrogen to freeze out impurities before the released F_2_ was premixed with neon or argon in a custom‐made stainless‐steel mixing chamber. The mixing chamber was connected to a self‐made matrix chamber by a stainless‐steel capillary. The gas mixture was condensed with laser‐ablated iridium atoms onto a gold‐plated mirror cooled to 6 K for neon and 12 K for argon using a closed‐cycle helium cryostat (Sumitomo Heavy Industries, RDK‐205D) inside the matrix chamber. For the laser‐ablation, the 1064 nm fundamental of a Nd:YAG laser (Continuum, Minilite II, 10 Hz repetition rate, 50–60 mJ pulse^−1^) was focused onto a rotating iridium metal target through a hole in the cold window. The infrared spectra were recorded on a Bruker Vertex 80v with 0.5 cm^−1^ resolution in the region 4000–450 cm^−1^ by using a liquid‐nitrogen‐cooled mercury cadmium telluride (MCT) detector. Matrix samples were annealed to different temperatures and irradiated by selected light‐emitting diode (LED) sources (OSLON 80 4+ PowerStar Circular 4 LED Arrays: *λ*=470±20 nm (blue), *λ*=365±10 nm (Qioptiq ML3 UV LED) and *λ*=278 nm (100 mW, AMPYR LED33UV278‐6060‐100), as well as a pulsed 266 nm Q‐switched solid‐state laser (CryLas 6FQSS266‐Q2‐OEM, 266/532 nm, 0.8 μJ @10 kHz).

UV‐vis spectra were recorded with a Perkin‐Elmer Lambda 850+ UV spectrometer in the range of 200–850 nm with a spectral resolution of 1.0 nm. The radiation of the spectrometer was directed into a quartz optical fiber of 2 m length, through a quartz lens inside the cryostat and passed two times over the matrix deposited on the cold gold mirror. A second quartz fiber collected the reflected radiation, and then directed it into the spectrometer.

Preparation of iridium hexafluoride followed procedures described in the literature.^[12a]^ It was prepared by heating iridium metal powder in a stainless‐steel autoclave with an excess of fluorine at 300 °C for about 8 h. Similar to our previous work on PtF_6_,^[21]^ the product IrF_6_ was stored in fluoroplastic (PFA) tube and trapped by liquid nitrogen. It was further purified by long pumping and its initial purity was monitored by IR spectroscopy. After purification, the gas sample was mixed by passing a stream of neon or argon gas through a cold PFA tube (−96 °C) containing the IrF_6_ sample and deposited on the matrix support for further measurements.

Initial quantum‐chemical structure optimizations of the molecules at density functional theory (DFT) level used the B3LYP^[23]^ hybrid functional in conjunction with the augmented triple‐*ζ* basis sets aug‐cc‐pVTZ for fluorine and the aug‐cc‐pVTZ‐PP^[24]^ valence basis and associated scalar‐relativistic pseudopotential (PP) for iridium. These calculations were performed using the Gaussian16 program package.^[25]^ All reasonable spin multiplicities have been considered. Subsequent structure optimizations as well as harmonic vibrational frequency analyses at the CCSD(T)^[16]^ (coupled‐cluster singles‐doubles with perturbational triples) level with aug‐cc‐pVTZ‐PP basis sets were carried out for the ground states of IrF_*n*_ (*n*=1–6) in the spin unrestricted ROHF‐UCCSD(T) open‐shell coupled cluster formalism using default frozen core settings as implemented in the Molpro 2019 software package.^[26]^ Due to the previously suggested significant SOC stabilization of IrF_5_,^[10]^ additional quasirelativistic all‐electron calculations using the exact two‐component (X2C) Hamiltonian at one‐ and two‐component (1c‐X2C and 2c‐X2C[^17^, ^27^]) DFT levels^[28]^ have been performed for all systems using Turbomole 7.5.0^[29]^ with x2c‐TZVPall‐2c all‐electron basis sets.^[30]^ Two‐electron SOC terms were approximated using the scaled‐nuclear‐spin‐orbit (SNSO)^[31]^ approach in its original parameterization by Böttcher.^[32]^ To optimize the IrF_4_ ⋅ F_2_ complex, dispersion corrections were included using Grimme's DFT‐D3^[33]^ scheme with Becke‐Johnson (BJ) damping^[34]^ for the B3LYP functional using aug‐cc‐pVTZ‐PP valence basis and the associated scalar‐relativistic pseudopotential (PP) for iridium.

## Conflict of interest

The authors declare no conflict of interest.

1

## Supporting information

As a service to our authors and readers, this journal provides supporting information supplied by the authors. Such materials are peer reviewed and may be re‐organized for online delivery, but are not copy‐edited or typeset. Technical support issues arising from supporting information (other than missing files) should be addressed to the authors.

Supporting Information

## Data Availability

The data that support the findings of this study are available from the corresponding author upon reasonable request.
