# Supplementary material for: Investigation of Molecular Iridium Fluorides IrF n (n=1–6): A Combined Matrix‐Isolation and Quantum‐Chemical Study
Source: Chemistry. 2022 Mar 18;28(22):e202104005. doi: 10.1002/chem.202104005 (PMC9310635; doi:10.1002/chem.202104005)
Supplement: Supplementary file 1 — Supporting Information [file CHEM-28-0-s001.pdf]

Chemistry–A European Journal

Supporting Information

Investigation of Molecular Iridium Fluorides IrF_n ($n = 1–6$): A Combined Matrix-Isolation and Quantum-Chemical Study

Yan Lu, Yetsedaw A. Tsegaw, Artur Wodyński, Lin Li, Helmut Beckers, Martin Kaupp, and Sebastian Riedel*

Contents

Table S1. Computed structures, electronic states of IrF_4......................................S4

Table S2. Computed structures, electronic states of IrF_5......................................S5

Table S3. Computed structures, electronic states of IrF_3......................................S6

Table S4. Computed structures, electronic states of IrF_2......................................S7

Table S5. Computed structures, electronic states and IR frequencies of IrF................S8

Table S6. Calculated IR frequencies of IrF_6 and IrF_7..S9

Table S7. Calculated IR frequencies of IrF_5...S10

Table S8. Calculated IR frequencies of IrF_4...S11

Table S9. Calculated IR frequencies of IrF_3...S12

Table S10. Calculated IR frequencies of IrF_2...S13

Table S11. Calculated IR frequencies of $IrF_4 \cdot F_2$ complex.....................................S14

Table S12. Computed thermochemical stability of iridium fluorides..........................S15

Table S13. Calculated T1 and D1 diagnostics values of iridium fluorides S16

Figure S1. Simplified scheme of Jahn-Teller distortion and spin-orbit coupling on the iridium 5d orbital splitting of IrF_5...S17

Figure S2. Computed structure of IrF_7 at B3LYP/aug-cc-pVTZ-PP level......................S17

Figure S3. Computed structures of iridium fluorides IrF_n at 2c-X2C-B3LYP level............S18

Figure S4. Computed structures of the quartet difluorine complex $IrF_4 \cdot F_2$....................S19

Figure S5. IR spectra in neon matrix at 6 K showing the photochemistry of IrF_6 (λ = 278 nm)..S20

Figure S6. IR spectra in argon matrix at 6 K showing the photochemistry of IrF_6 (λ = 278 nm)..S21

Figure S7. IR spectra in neon matrix at 6 K showing the photochemistry of IrF_6 (λ = 365 nm and 470 nm)...S22

Figure S8. IR spectra in argon matrix at 6 K showing the photochemistry of IrF_6 (λ = 365 nm and 470 nm) S23

Figure S9. IR spectra in argon matrix at 6 K showing the photochemistry of IrF_6 (λ = 365 nm and annealing to 15 K) S24

Figure S10. IR spectra in neon matrix at 6 K showing the photochemistry of IrF_6 ($\lambda = 278$ nm and 266 nm)……………………………………………………………………………..S25

Calculated atomic coordinates of species at scalar relativistic levels (with PP)………....S26

Calculated atomic coordinates at X2C level ………………………………...…...........S37

References……………………………………………………………………………..S40

Table S1a. Electronic states, structural parameters (pm, deg), electronic energy differences (kJ mol^{-1}) of selected states of IrF_4 at scalar-relativistic pseudopotential levels.

	CCSD(T)[a]				B3LYP/aT-PP		
Electronic state (Sym.)	Bond lengths [pm] Ir–F/Ir–F'	Angle [°] F–Ir–F	ΔE	ΔE + ΔZPE	Bond lengths [pm] Ir–F/Ir–F'	Angle [°] F–Ir–F	ΔE + ΔZPE
$^2B_2$ (D_{2d})	185.3	124.1	154.6	149.5	186.3	124.3	144.5
$^2B_{1g}$ (D_{2h})	–	–	–	–	180.7/186.0	90.0	102.2
$^2B_{3g}$ (D_{2h})	179.9/185.1	90.0	109.3	108.9	–	–	–
$^4B_{2g}$ (D_{4h})	183.4	90.0	0.0	0.0	184.4	90.0	0.0
$^6A_1$ (T_d)	–	–	–	–	193.3	109.5	241.1

[a]aug-cc-pVTZ-PP basis sets.

Table S1b. Electronic states and structural parameters (pm, deg) of selected states of IrF_4 at one- and two-component all-electron X2C levels.

	1c-X2C-B3LYP all-electron		2c-X2C-B3LYP all-electron	
Electronic state (Sym.)	Bond lengths [pm] Ir–F	Angle [°] F–Ir–F	Bond lengths [pm] Ir–F	Angle [°] F–Ir–F
$^4B_{2g}$ (D_{4h})	184.5	90	184.8	90

Table S2a. Electronic states, structural parameters (pm, deg), electronic energy differences (kJ mol^{-1}) of selected states of IrF_5 at scalar-relativistic pseudopotential levels.

Electronic state (Sym.)	Parameter	CCSD(T)[a]	B3LYP/aT-PP	$\Delta E_{CCSD(T)}$	$\Delta E_{CCSD(T)}$ + ΔZPE	ΔE_{B3LYP} + ΔZPE
$^1A_1'$ (D_{3h})	$d_{Ir\text{-}F(ax)}$	192.6	194.3	41.9	42.2	61.1
	$d_{Ir\text{-}F(eq)}$	178.6	179.6			
$^1A_1$ (C_{4v})	$d_{Ir\text{-}F(ax)}$	186.8	188.0	47.0	47.3	53.3
	$d_{Ir\text{-}F(eq)}$	184.0	185.1			
	$\measuredangle_{F(ax)\text{-}Ir\text{-}F(eq)}$	91.3	91.5			
	$\measuredangle_{F(eq)\text{-}Ir\text{-}F(eq)}$	90.0	90.0			
$^3B_1$ (C_{2v})	$d_{Ir\text{-}F(1)}$	182.6	183.5	0.0	0.0	0.0
	$d_{Ir\text{-}F(2)}$	188.0	189.2			
	$d_{Ir\text{-}F(3)}$	182.7	184.1			
	$\measuredangle_{F(1)\text{-}Ir\text{-}F(2)}$	91.2	91.5			
	$\measuredangle_{F(2)\text{-}Ir\text{-}F(3)}$	89.8	89.8			
	$\measuredangle_{F(1)\text{-}Ir\text{-}F(3)}$	98.2	98.5			
$^5B_1$ (C_{4v})	$d_{Ir\text{-}F(ax)}$	195.3	197.8	24.0	24.2	14.9
	$d_{Ir\text{-}F(eq)}$	183.8	185.0			
	$\measuredangle_{F(ax)\text{-}Ir\text{-}F(eq)}$	96.0	95.6			
	$\measuredangle_{F(eq)\text{-}Ir\text{-}F(eq)}$	89.4	89.4			

[a]aug-cc-pVTZ-PP basis sets.

Table S2b. Electronic states, structural parameters (pm, deg), electronic energy differences (kJ mol^{-1}) of selected states of IrF_5 at one- and two-component all-electron X2C levels.

Level	Sym.	Multiplicity	Functional[a]	E_{B3LYP} + ΔZPE	$d_{Ir\text{-}F(ax)}$ [pm]	$d_{Ir\text{-}F(eq1)}$ [pm]	$d_{Ir\text{-}F(eq2)}$ [pm]	$\measuredangle_1$ [deg]	$\measuredangle_2$ [deg]
1c-X2C	C_{4v}	5	B3LYP	15.4	197.8	185.0	–	95.7	–
1c-X2C	C_{2v}	3	B3LYP	0	183.5	184.1	189.2	91.5	98.5
2c-X2C	C_{4v}	(3)	B3LYP	-[b]	185.9	186.4	–	94.3	–

[a]x2c-TZVPall-2c all-electron basis sets.

[b]2c-X2C computations did not converge for quintet 1c-X2C guess. Comparison of quintet and triplet configurations is not possible.

Table S3a. Electronic states, structural parameters (pm, deg), electronic energy differences (kJ mol^{-1}) of selected states of IrF_3 at scalar-relativistic pseudopotential levels.

	CCSD(T)[a]				B3LYP/aT-PP		
Electronic state (Sym.)	Bond lengths [pm] Ir–F/Ir–F'	Angle [°] F–Ir–F	ΔE	ΔE + ΔZPE	Bond lengths [pm] Ir–F/Ir–F'	Angle [°] F–Ir–F	ΔE + ΔZPE
$^1A_1'$ (D_{3h})	181.1	120.0	37.0	37.1	181.8	120.0	44.2
$^3B_1$ (C_{2v})	183.6/185.1	164.7	0.0	0.0	184.4/185.9	164.3	0.0

[a]aug-cc-pVTZ-PP basis sets.

Table S3b. Electronic states and structural parameters (pm, deg) of selected states of IrF_3 at one- and two-component all-electron X2C levels.

	1c-X2C-B3LYP all-electron		2c-X2C-B3LYP all-electron	
Electronic state (Sym.)	Bond lengths [pm] Ir–F/Ir–F'	Angle [°] F–Ir–F	Bond lengths [pm] Ir–F/Ir–F'	Angle [°] F–Ir–F
$^3B_1$ (C_{2v})	184.4/185.9	164.3	185.9/186.4	165.2

Table S4a. Electronic states, structural parameters (pm, deg), electronic energy differences (kJ mol^{-1}) of selected states of IrF_2 at scalar-relativistic pseudopotential levels.

	CCSD(T)[a]				B3LYP/aT-PP		
Electronic state (Sym.)	Bond lengths [pm] Ir–F	Angle [°] F–Ir–F	ΔE	ΔE + ΔZPE	Bond lengths [pm]	Angle [°] F–Ir–F	ΔE + ΔZPE
$^2A_2$ (C_{2v})	–	–	–	–	179.8	166.2	62.7
$^4\Delta_g$ ($D_{\infty h}$)	184.9	180.0	0.0	0.0	185.4	180.0	0.0
$^4\Sigma_g^-$ ($D_{\infty h}$)	184.7	180.0	6.1	6.1	–	–	–
$^4\Phi_g$ ($D_{\infty h}$)	190.4	180.0	71.5	70.9	–	–	–

[a]aug-cc-pVTZ-PP basis sets.

Table S4b. Electronic states and structural parameters (pm, deg) of selected states of IrF_2 at one- and two-component all-electron X2C levels.

	1c-X2C-B3LYP all-electron		2c-X2C-B3LYP all-electron	
Electronic state (Sym.)	Bond lengths [pm] Ir–F	Angle [°] F–Ir–F	Bond lengths [pm] Ir–F	Angle [°] F–Ir–F
$^4\Delta_g$ ($D_{\infty h}$)	185.3	180.0	186.0	180.0

Table S5a. Electronic states, structural parameters (pm), electronic energy differences (kJ mol^{-1}) and predicted frequencies (cm^{-1}) of selected states of IrF at scalar-relativistic pseudopotential levels.

	CCSD(T)^a				B3LYP/aT-PP		
Electronic state (Sym.)	Bond lengths [pm] Ir–F	Freq.	ΔE	ΔE + ΔZPE	Bond lengths [pm] Ir–F	Freq.	ΔE + ΔZPE
$^1\Sigma^+$ ($C_{\infty v}$)	181.7	638.0	123.1	123.2	180.9	656.4	152.8
$^3\Sigma^-$ ($C_{\infty v}$)	182.9	614.8	0.4	0.3	182.2	618.4	17.1
$^3\Phi$ ($C_{\infty v}$)	186.1	632.6	0.0	0.0	186.4	628.5	0.0
$^3\Delta$ ($C_{\infty v}$)	190.7	615.7	8.4	8.3	–	–	–

^{a}aug-cc-pVTZ-PP basis sets.

Table S5b. Electronic states, structural parameters (pm) and predicted frequencies (cm^{-1}) of selected states of IrF at one- and two-component all-electron X2C levels.

	1c-X2C-B3LYP all-electron		2c-X2C-B3LYP all-electron	
Electronic state (Sym.)	Bond lengths [pm] Ir–F	Freq.	Bond lengths [pm] Ir–F	Freq.
$^3\Phi$ ($C_{\infty v}$)	186.1	639	186.2	652

Table S6. Calculated IR frequencies of IrF_6 and IrF_7.

Molecule	Electronic state (Sym.)	mode	B3LYP/aT-PP	1c-X2C-B3LYP	2c-X2C-B3LYP	Ref.[1]
		T_{1u}	711.6 (177)	715 (174)	716 (149)[a]	
		T_{1u}	711.6 (177)	715 (174)	716 (153)[a]	
		T_{1u}	711.6 (177)	715 (174)	715 (149)[a]	
		A_{1g}	706.8 (0)	709 (0)	707 (1)	
		E_g	647.1 (0)	648 (0)	656 (0)	
		E_g	647.1 (0)	648 (0)	654 (0)	
		T_{1u}	278.5 (14)	278 (15)	302 (7)[a]	
IrF_6	$^4A_{1g}$ (O_h)	T_{1u}	278.5 (14)	278 (15)	299 (8)[a]	
		T_{1u}	278.5 (14)	278 (15)	299 (10)[a]	
		T_{2g}	262.1 (0)	267 (0)	278 (0)	
		T_{2g}	262.1 (0)	267 (0)	276 (0)	
		T_{2g}	262.1 (0)	267 (0)	274 (0)	
		T_{2u}	204.3 (0)	207 (0)	238 (0)	
		T_{2u}	204.3 (0)	207 (0)	235 (0)	
		T_{2u}	204.3 (0)	207 (0)	235 (0)	
		A_2''	714.4 (153)	718 (149)	718 (122)	691.0
		A_1'	681.4 (0)	683 (0)	681 (1)	657.4
		E_1'	661.9 (113)	662 (110)	665 (88)[a]	635.4
		E_1'	661.9 (113)	662 (110)	659 (94)[a]	
		A_1'	628.2 (0)	628 (0)	636 (0)	606.7
		E_2'	562.5 (0)	560 (0)	564 (0)[a]	542.6
		E_2'	562.5 (0)	560 (0)	563 (0)[a]	
		E_2'	472.8 (0)	471 (0)	479 (0)[a]	456.1
		E_2'	472.8 (0)	471 (0)	477 (0)[a]	
IrF_7	$^3A_1'$ (D_{5h})	E_1'	335.3 (14)	331 (13)	347 (5)[a]	319.2
		E_1'	335.3 (14)	331 (13)	346 (5)[a]	
		A_2''	302.7 (13)	300 (13)	325 (7)	292.8
		E_1''	260.3 (0)	262 (0)	276 (0)[a]	255.2
		E_1''	260.3 (0)	262 (0)	273 (0)[a]	
		E_1'	237.3 (3)	237 (4)	262 (1)[a]	227.8
		E_1'	237.3 (3)	237 (4)	260 (2)[a]	
		E_2''	94.8 (0)	102 (0)	154 (0)[a]	91.3
		E_2''	94.8 (0)	102 (0)	152 (0)[a]	

[a]Observed splitting for the degenerate modes in the 2c-calculation is due to numerical errors resulting from the use of C_1 symmetry. Frequencies in cm^{-1}, intensities are shown in parentheses in km mol^{-1}.

Table S7. Calculated IR frequencies of IrF_5.

Electronic state (Sym.)	mode	B3LYP/aT-PP	1c-X2C-B3LYP	2c-X2C-B3LYP	CCSD(T)[a]	Ref.[2]
	A_1	716.8 (16)	720 (16)	–	725.8	–
	B_2	702.4 (177)	706 (177)	–	724.4	–
	A_1	692.2 (15)	695 (15)	–	710.4	–
	B_1	662.3 (194)	667 (190)	–	682.5	–
	A_1	633.6 (0)	637 (0)	–	652.5	–
	B_1	261.8 (8)	276 (8)	–	263.5	–
$^3B_1$ (C_{2v})	A_1	247.1 (12)	247 (12)	–	254.1	–
	B_2	219.5 (13)	240 (13)	–	229.8	–
	A_2	215.3 (0)	257 (0)	–	223.1	–
	A_1	162.4 (1)	164 (1)	–	168.0	–
	B_2	112.8 (1)	130 (1)	–	125.0	–
	B_1	67.5 (3)	84 (3)	–	90.2	–
	E	704.4 (174)	708 (173)	–	724.1	702 (173)
	E	704.4 (174)	708 (173)	–	724.1	702 (173)
	A_1	693.4 (3)	697 (3)	–	710.7	693 (1)
	B_2	630.0 (0)	633 (0)	–	647.6	628 (0)
	A_1	524.4 (51)	524 (48)	–	566.2	526 (21)
	B_1	273.9 (0)	278 (0)	–	280.0	267 (0)
$^5B_1$ (C_{4v})	E	247.1 (9)	248 (9)	–	250.5	239 (9)
	E	247.1 (9)	248 (9)	–	250.5	239 (9)
	A_1	215.1 (10)	214 (10)	–	219.2	218 (10)
	E	173.2 (1)	173 (1)	–	179.3	172 (1)
	E	173.2 (1)	173 (1)	–	179.2	172 (1)
	B_2	135.0 (0)	144 (0)	–	137.2	139 (0)
Spin-orbit (C_{4v})	A_1	–	–	690 (186)	–	702 (173)
	A_1	–	–	690 (187)	–	702 (173)
	A_1	–	–	696 (1)	–	693 (1)
	A_1	–	–	638 (37)	–	628 (0)
	A_1	–	–	608 (0)	–	526 (21)
	A_1	–	–	266 (0)	–	267 (0)
	A_1	–	–	245 (10)	–	239 (9)
	A_1	–	–	244 (10)	–	239 (9)
	A_1	–	–	233 (14)	–	218 (10)
	A_1	–	–	163 (0)	–	172 (1)
	A_1	–	–	152 (2)	–	172 (1)
	A_1	–	–	150 (1)	–	139 (0)

[a]aug-cc-pVTZ-PP basis sets. Frequencies in cm^{-1}, intensities are shown in parentheses in km mol^{-1}.

Table S8. Calculated IR frequencies of IrF_4.

Electronic state (Sym.)	mode	B3LYP/aT-PP	1c-X2C-B3LYP	2c-X2C-B3LYP	CCSD(T)[a]
$^2B_2$ (D_{2d})	B_2	680.3 (141)	–	–	696.4
	A_1	677.3 (0)	–	–	690.6
	E	603.4 (80)	–	–	621.1
	E	603.4 (80)	–	–	620.6
	A_1	261.3 (0)	–	–	261.7
	B_2	215.6 (17)	–	–	208.7
	E	73.3 (14)	–	–	49.6
	E	73.3 (14)	–	–	45.7
	B_1	28.1 (0)	–	–	37.2
$^2B_{1g}$ (D_{2h})	B_{2u}	739.9 (191)	–	–	–
	A_g	723.0 (0)	–	–	–
	B_{1u}	683.9 (0)	–	–	–
	A_g	636.7(0)	–	–	–
	B_{3u}	270.5(5)	–	–	–
	B_{3g}	224.5(0)	–	–	–
	B_{1u}	217.9(16)	–	–	–
	B_{3u}	182.3(1)	–	–	–
	B_{2u}	168.9(19)	–	–	–
$^4B_{2g}$ (D_{4h})	E_u	710.6 (185)	716 (183)	715 (161)[b]	727.9
	E_u	710.6 (185)	716 (183)	713 (168)[b]	727.9
	A_{1g}	706.1 (0)	710 (0)	704 (1)	722.0
	B_{1g}	652.8 (0)	655 (0)	657 (0)	669.8
	B_{2g}	264.3 (0)	268 (0)	277 (0)	270.2
	E_u	244.7 (8)	245 (8)	263 (5)[b]	247.6
	E_u	244.7 (8)	245 (8)	261 (6)[b]	247.6
	A_{2u}	200.4 (11)	198 (11)	224 (10)	203.2
	B_{2u}	131.5 (0)	129 (0)	165 (0)	132.1
$^2B_{3g}$ (D_{2h})	B_{2u}	–	–	–	753.6
	A_g	–	–	–	733.1
	B_{1u}	–	–	–	699.8
	A_g	–	–	–	644.9
	B_{3u}	–	–	–	272.6
	B_{3g}	–	–	–	221.1
	B_{1u}	–	–	–	210.8
	B_{3u}	–	–	–	182.8
	B_{2u}	–	–	–	164.1

[a]aug-cc-pVTZ-PP basis sets. [b]Observed splitting for the degenerate modes in the 2c-calculation is due to numerical errors resulting from the use of C_1 symmetry. Frequencies in cm^{-1}, intensities are shown in parentheses in km mol^{-1}.

Table S9. Calculated IR frequencies of IrF_3.

Electronic state (Sym.)	mode	B3LYP/aT-PP	1c-X2C-B3LYP	2c-X2C-B3LYP	CCSD(T)^a
$^1A_1'$ (D_{3h})	A_1'	718.0 (0)	–	–	726.8
	E'	699.8 (126)	–	–	712.4
	E'	699.8 (126)	–	–	712.3
	E'	170.8 (3)	–	–	169.1
	E'	170.8 (3)	–	–	165.6
	A_2''	92.9 (4)	–	–	165.0
$^3B_1$ (C_{2v})	A_1	694.4 (1)	700 (0)	680 (9)	710.6
	B_2	691.4 (202)	699 (200)	689 (181)	708.3
	A_1	650.4 (61)	655 (62)	651 (42)	663.9
	B_1	194.4 (5)	196 (5)	198 (7)	197.5
	A_1	187.3 (8)	188 (8)	197 (7)	194.7
	B_2	119.2 (5)	122 (5)	167 (5)	169.5

^{a}aug-cc-pVTZ-PP basis sets. Frequencies in cm^{-1}, intensities are shown in parentheses in km mol^{-1}.

Table S10. Calculated IR frequencies of IrF_2.

Electronic state (Sym.)	mode	B3LYP/aT-PP	1c-X2C-B3LYP	2c-X2C-B3LYP	CCSD(T)[a]
$^4\Delta_g$ ($D_{\infty h}$)	Σ_u^+	700.5 (178)	709 (176)	701 (158)	709.8
	Σ_g^+	666.1 (0)	673 (0)	665 (0)	671.2
	Π_u	156.6 (5)	158 (5)	182 (4)[b]	159.0
	Π_u	156.6 (5)	158 (5)	179 (4)[b]	159.0
$^4\Phi_g$ ($D_{\infty h}$)	Σ_u^+	–	–	–	643.0
	Σ_g^+	–	–	–	634.4
	Π_u	–	–	–	208.4
	Π_u	–	–	–	109.3
$^4\Sigma_g^-$ ($D_{\infty h}$)	Σ_u^+	–	–	–	687.5
	Σ_g^+	–	–	–	676.5
	Π_u	–	–	–	173.0
	Π_u	–	–	–	173.0
$^2A_2$ (C_{2v})	B_2	746.2 (176)	–	–	–
	A_1	712.8 (3)	–	–	–
	A_1	64.6 (2)	–	–	–

[a]aug-cc-pVTZ-PP basis sets. [b]Observed splitting for the degenerate modes in the 2c-calculation is due to numerical errors resulting from the use of C_1 symmetry. Frequencies in cm^{-1}, intensities are shown in parentheses in km mol^{-1}.

Table S11. Calculated IR frequencies of difluorine complex $IrF_4{\cdot}F_2$.[a]

		$\Delta E_{B3LYP}+\Delta ZPE$ [kJ mol^{-1}]	Freq.		$\Delta E_{B3LYP}+\Delta ZPE$ [kJ mol^{-1}]	Freq.
			1051.9 (0)			1050.9 (0)
			740.0 (183)			710.6 (182)
			722.8 (0)			710.3 (178)
			682.2 (180)			706.1 (0)
			635.3 (0)			652.6 (0)
			270.7 (5)			264.4 (0)
			218.2 (0)			244.8 (7)
side-on	doublet	102.5	217.7 (16)	quartet	0.0	244.7 (7)
			182.2 (1)			200.5 (12)
			169.6 (18)			131.4 (0)
			36.0 (0)			38.8 (0)
			35.0 (0)			29.9 (0)
			13.0 (0)			13.8 (0)
			11.0 (0)			8.6 (0)
			4.8 (0)			8.4 (0)
			967.1 (66)			1016.9 (14)
			742.2 (185)			712.1 (181)
			724.0 (1)			712.1 (181)
			684.7 (178)			707.1 (1)
			637.8 (0)			654.3 (0)
			270.8 (3)			264.9 (0)
			245.0 (0)			245.0 (7)
end-on	doublet	102.6	218.4 (16)	quartet	1.5	245.0 (7)
			182.2 (0)			199.9 (10)
			169.6 (19)			131.5 (0)
			70.0 (0)			34.9 (0)
			59.4 (0)			30.3 (0)
			41.7 (1)			30.3 (0)
			21.8 (0)			14.3 (0)
			20.3 (0)			14.3 (0)

[a]B3LYP/aug-cc-pVTZ-PP-D3 level. Frequencies in cm^{-1}, intensities are shown in parentheses in km mol^{-1}.

Table S12. Computed thermochemical stability of iridium fluorides in kJ mol^{-1} at different levels of theory.

Reaction	B3LYP/aT-PP		1c-X2C-B3LYP[a]		2c-X2C-B3LYP[a]		CCSD(T)[b]	
	ΔE + ΔZPE	ΔH	ΔE + ΔZPE	ΔH	ΔE + ΔZPE	ΔH	ΔE + ΔZPE (B3LYP)[c]	ΔH[d]
$IrF_6 \rightarrow IrF_4 + F_2$	336.4	339.9	321.9	325.5	312.7	316.6	308.9	312.5
$IrF_6 \rightarrow IrF_5 + F$	329.6	334.8	302.7	305.2	256.0	260.5	310.1	315.4
$IrF_5 \rightarrow IrF_3 + F_2$	394.3	396.3	397.1	402.0	421.3	424.6	374.4	376.5
$IrF_5 \rightarrow IrF_4 + F$	155.8	157.8	166.3	171.1	204.0	207.0	145.0	146.9
$IrF_4 \rightarrow IrF_2 + F_2$	544.6	547.8	528.1	531.2	507.2	510.6	526.2	529.4
$IrF_4 \rightarrow IrF_3 + F$	387.5	391.2	378.0	381.7	364.6	368.5	375.7	379.4
$IrF_3 \rightarrow IrF + F_2$	654.2	656.3	638.4	640.5	607.2	609.5	654.1	656.2
$IrF_3 \rightarrow IrF_2 + F$	306.2	309.3	297.2	300.3	289.9	293.1	296.8	299.8
$IrF_2 \rightarrow Ir + F_2$	730.8	733.1	701.9	704.3	681.7	684.3	716.9	719.2
$IrF_2 \rightarrow IrF + F$	497.1	499.7	488.3	490.9	464.5	467.3	503.6	506.2
$IrF \rightarrow Ir + F$	382.7	386.0	360.8	364.1	364.5	367.9	359.6	362.9

[a]x2c-TZVPall-2c basis sets. [b]aug-cc-pVTZ-PP basis sets. [c]Using B3LYP zero point energy corrections for the electronic energies at CCSD(T) level. [d]The enthalpies at CCSD(T) level were calculated by adding the enthalpy corrections (B3LYP) to electronic energy changes.

Table S13. T1 and D1 diagnostics values of IrF_n (n = 1–6) calculated at the CCSD(T)/aug-cc-pVTZ-PP level of theory. These values are smaller than the T ≥ 0.05 and D ≥ 0.15 limits proposed in the literature for transition metal compounds,[3] so no multiconfiguration calculations were employed for these species.

**Species**	**T1**	**D1**
IrF_6	0.03425218	0.12759613
IrF_5	0.03238256	0.12958705
IrF_4	0.03527559	0.11454362
IrF_3	0.03148315	0.09254815
IrF_2	0.02993183	0.08749521
IrF	0.02846659	0.08275391

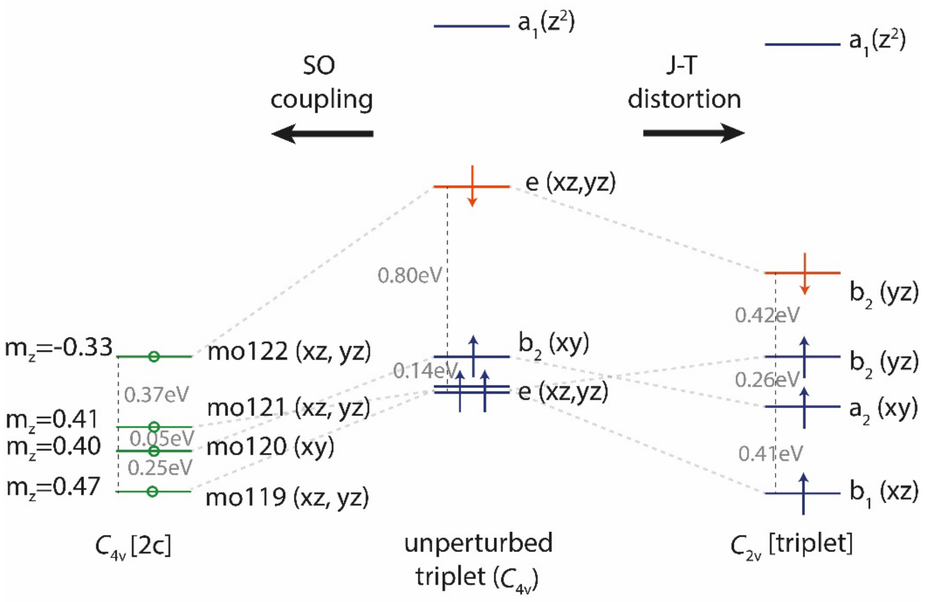

Figure S1. Simplified scheme of Jahn-Teller (JT) distortion and spin-orbit coupling (SOC) on the iridium 5d orbital splitting of triplet pyramidal IrF_5. The energy levels for the unperturbed C_{4v} triplet state were modeled from an unrestricted calculation by spatial averaging of the b_1 and b_2 levels of a C_{2v}-symmetrical wave function to the e-level that is shown.

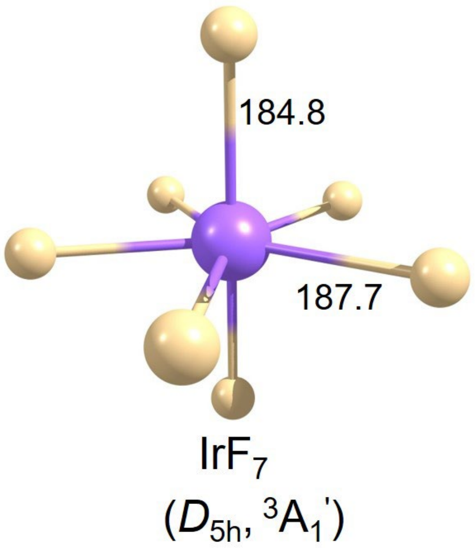

Figure S2. Computed structures of molecular IrF_7 at the B3LYP/aug-cc-pVTZ-PP level. Selected bond lengths (pm) are shown.

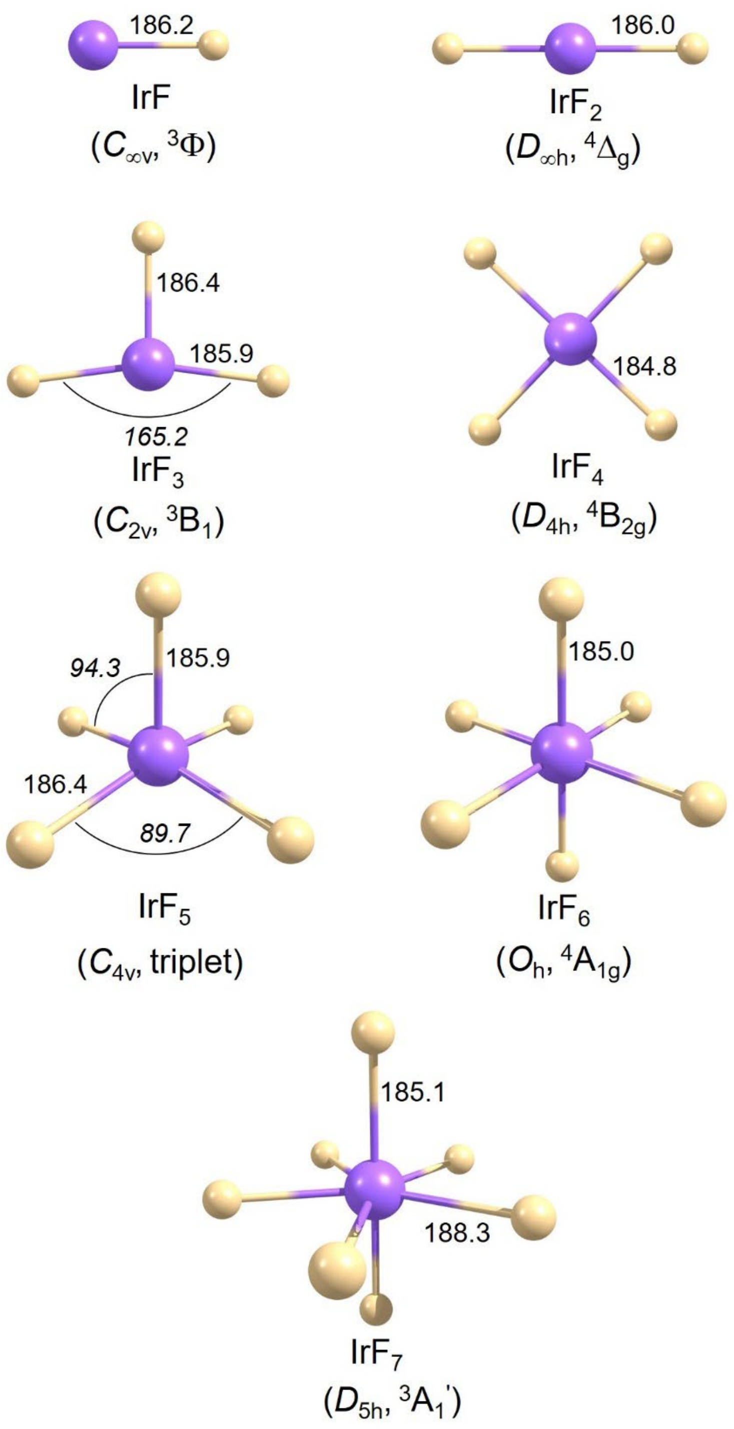

Figure S3. Computed structures of molecular iridium fluorides IrF_n (n = 1–7) at 2c-X2C-B3LYP/x2c-TZVPall-2c level. Selected bond lengths (pm) and angles (º, in *italics*) are shown.

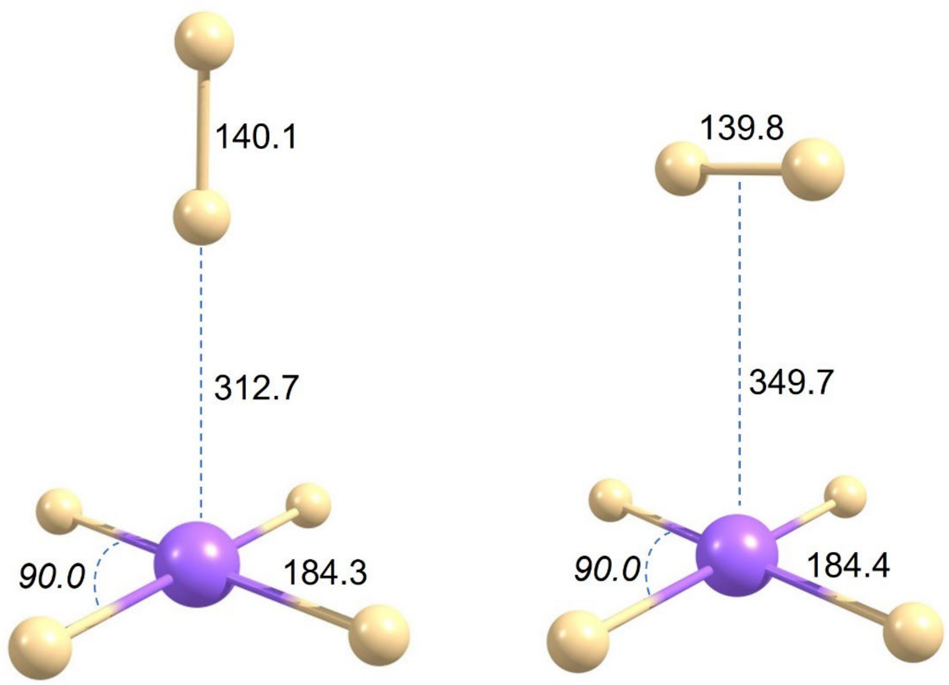

Figure S4. Computed structures of end-on and side-on structures of the quartet difluorine complex $IrF_4{\cdot}F_2$ at the B3LYP/aug-cc-pVTZ-PP-D3 level. Bond lengths [pm] and angles [º, in *italics*] are shown.

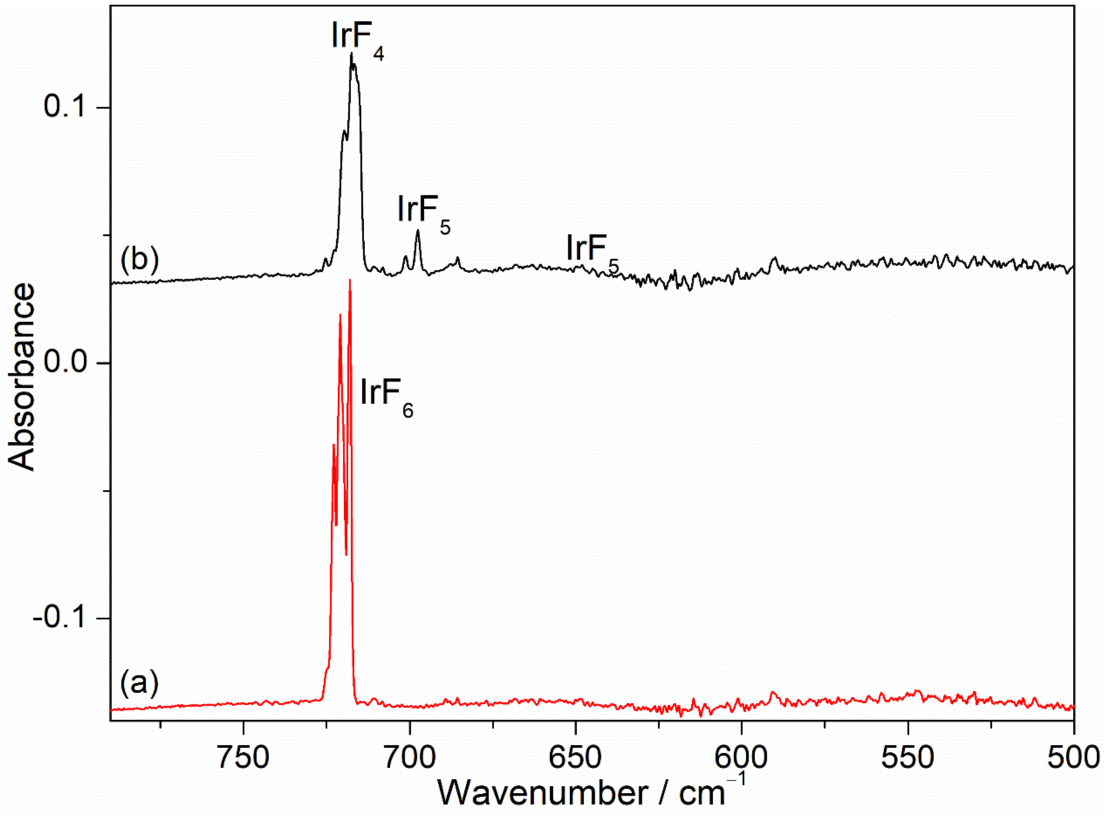

Figure S5. IR spectra in neon matrix at 6 K showing the photochemistry of IrF_6. (a) Spectrum of IrF_6 obtained after deposition for 15 min, (b) IR spectrum obtained after λ = 278 nm irradiation for 90 min.

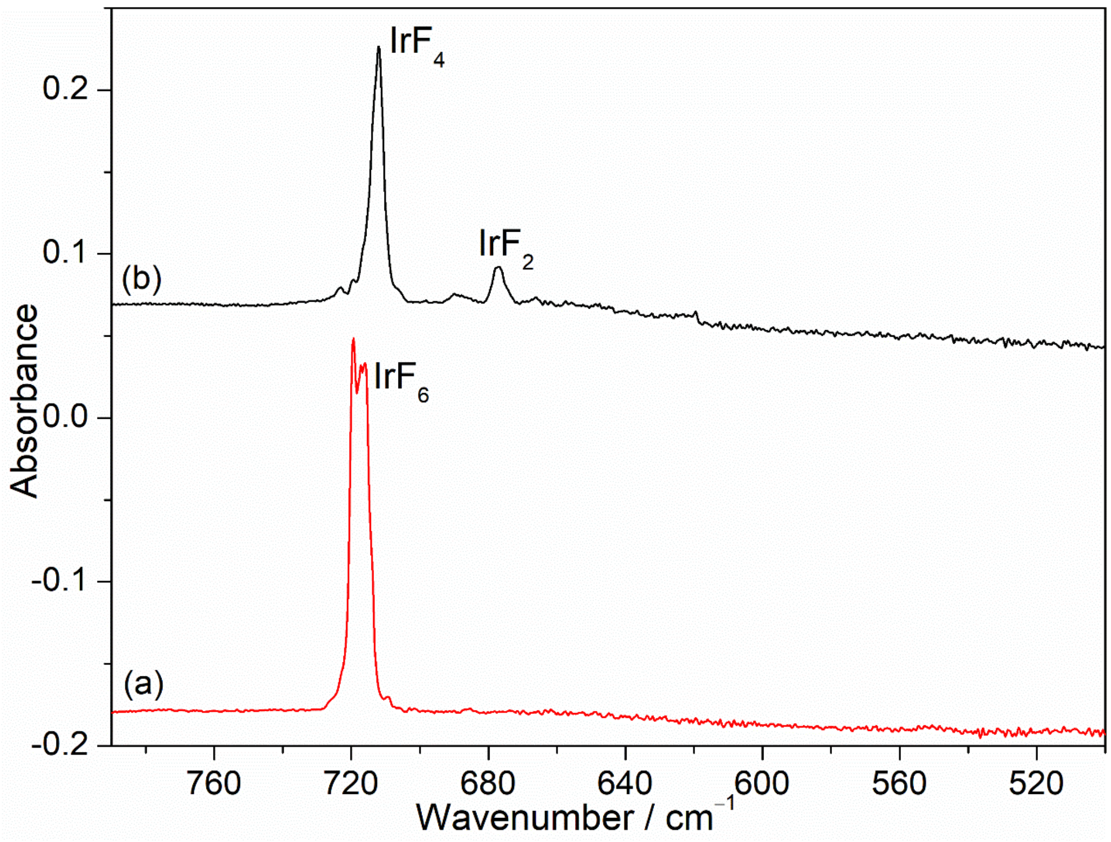

Figure S6. IR spectra in argon matrix at 6 K showing the photochemistry of IrF_6. (a) Spectrum of IrF_6 obtained after deposition for 25 min, (b) IR spectrum obtained after λ = 278 nm irradiation for 110 min.

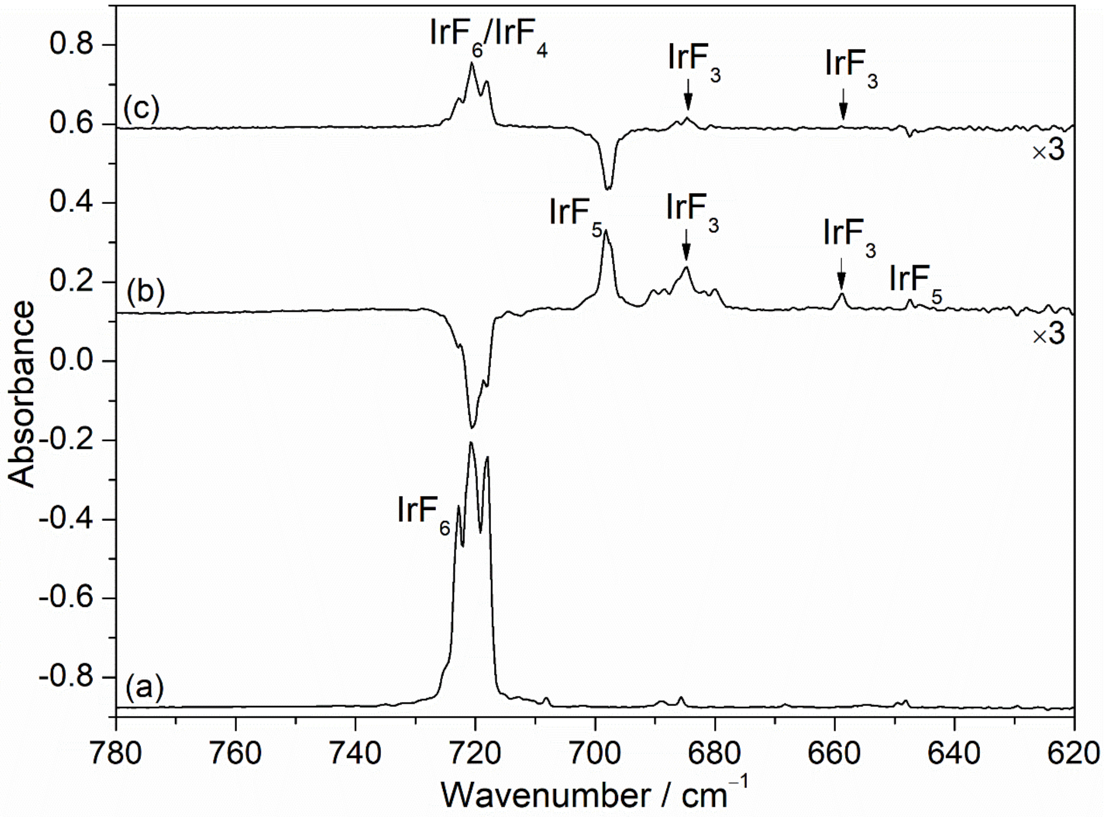

Figure S7. IR spectra in neon matrix at 6 K showing the photochemistry of IrF_6. (a) Spectrum of IrF_6 obtained after deposition for 30 min, (b) difference IR spectrum obtained after $\lambda = 365$ nm irradiation for 40 min, and (c) subsequent irradiation of the same matrix at $\lambda = 470$ nm irradiation for 15 min.

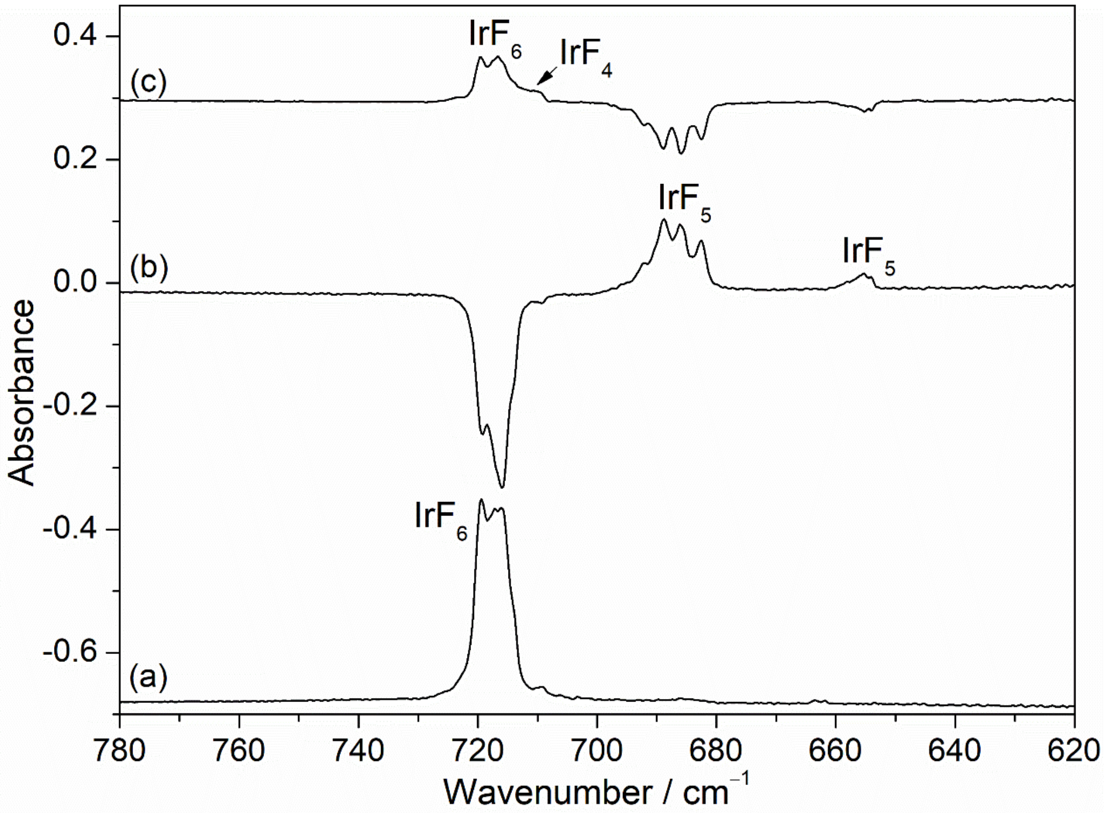

Figure S8. IR spectra in argon matrix at 6 K showing the photochemistry of IrF_6. (a) Spectrum of IrF_6 obtained after deposition for 30 min, (b) difference IR spectrum obtained after $\lambda = 365$ nm irradiation for 60 min, and (c) subsequent irradiation of the same matrix at $\lambda = 470$ nm for 45 min.

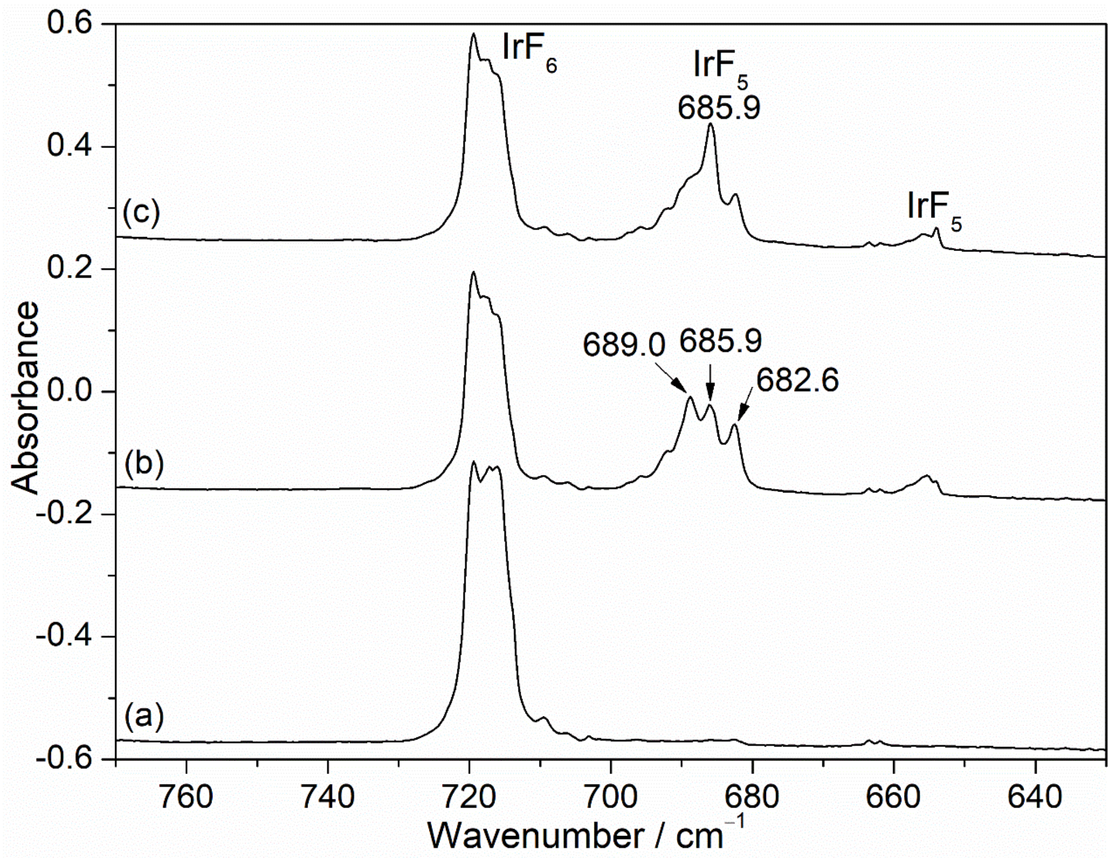

Figure S9. IR spectra in argon matrix at 6 K showing the photochemistry of IrF_6. (a) Spectrum of IrF_6 obtained after deposition for 20 min, (b) IR spectrum obtained after λ = 365 nm irradiation for 90 min, and (c) subsequent annealing to 15 K.

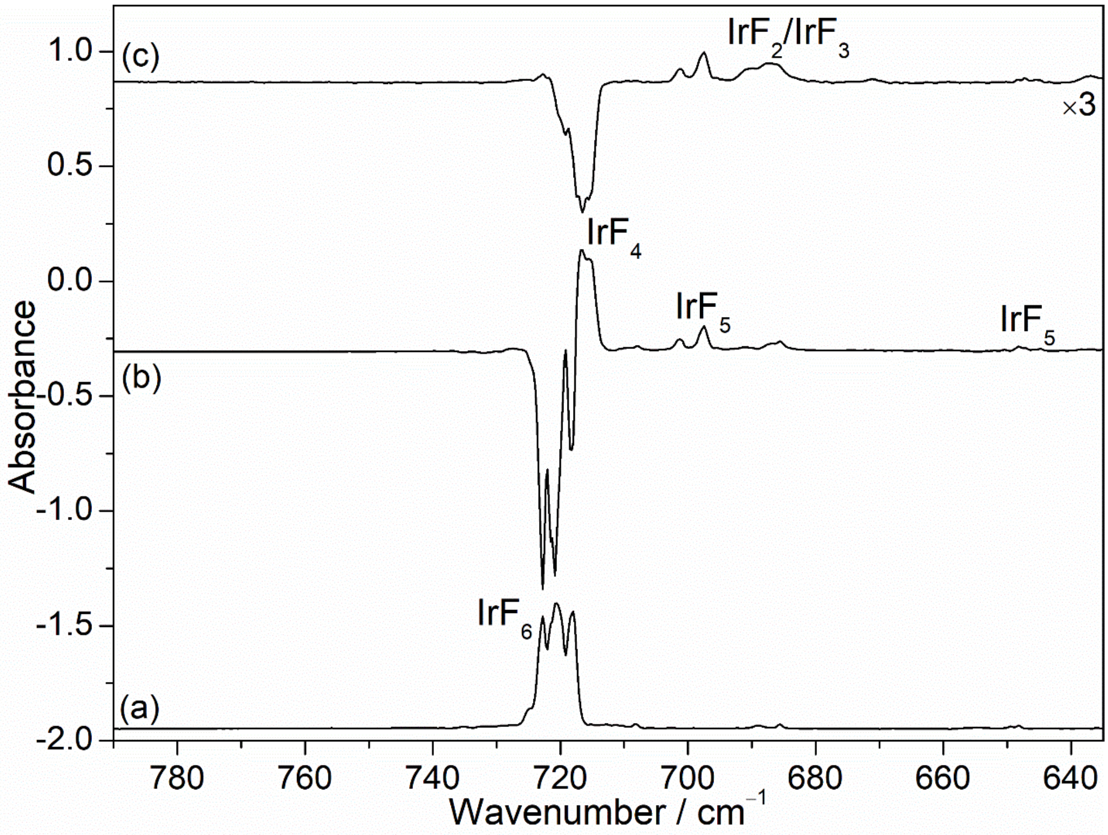

Figure S10. IR spectra in neon matrix at 6 K showing the photochemistry of IrF_6. (a) Spectrum of IrF_6 obtained after deposition for 20 min, (b) difference IR spectrum obtained after $\lambda = 278$ nm irradiation for 180 min, and (c) subsequent irradiation of the same matrix at $\lambda = 266$ nm irradiation for 25 min.

Calculated atomic coordinates (in Å) of species for optimized structures at B3LYP/aug-cc-pVTZ-PP level.

IrF_7 $^3A_1'$ (D_{5h})

Ir	0.000000000	0.000000000	0.000000000
F	0.000000000	0.000000000	1.848213000
F	0.000000000	1.876680000	0.000000000
F	-1.784829000	0.579926000	0.000000000
F	0.000000000	0.000000000	-1.848213000
F	-1.103085000	-1.518266000	0.000000000
F	1.103085000	-1.518266000	0.000000000
F	1.784829000	0.579926000	0.000000000

IrF_6 $^4A_{1g}$ (O_h)

Ir	0.00000000	0.00000000	0.00000000
F	0.00000000	0.00000000	1.84598900
F	0.00000000	1.84598900	0.00000000
F	-1.84598900	0.00000000	0.00000000
F	0.00000000	0.00000000	-1.84598900
F	0.00000000	-1.84598900	0.00000000
F	1.84598900	0.00000000	0.00000000

IrF_5 $^1A_1'$ (D_{3h})

Ir	0.00000000	0.00000000	0.00000000
F	0.00000000	1.79587400	0.00000000
F	0.00000000	0.00000000	1.94339800
F	-1.55527200	-0.89793700	0.00000000
F	1.55527200	-0.89793700	0.00000000
F	0.00000000	0.00000000	-1.94339800

IrF_5 $^1A_1$ (C_{4v})

Ir	0.00000000	0.00000000	0.12427700
F	0.00000000	0.00000000	-1.75525400
F	0.00000000	1.85073600	0.17299900
F	-1.85073600	0.00000000	0.17299900
F	0.00000000	-1.85073600	0.17299900
F	1.85073600	0.00000000	0.17299900

IrF_5 $^3B_1$ (C_{2v})

Ir	0.00000000	0.00000000	0.08782400
F	0.00000000	0.00000000	-1.74758300
F	0.00000000	1.82014900	0.36097100
F	-1.89125800	0.00000000	0.13713100
F	0.00000000	-1.82014900	0.36097100
F	1.89125800	0.00000000	0.13713100

IrF_5 $^5B_1$ (C_{4v})

Ir	0.00000000	0.00000000	0.09091000
F	0.00000000	0.00000000	-1.88715500
F	0.00000000	1.84025600	0.27734200
F	-1.84025600	0.00000000	0.27734200
F	0.00000000	-1.84025600	0.27734200
F	1.84025600	0.00000000	0.27734200

IrF_4 $^2B_2$ (D_{2d})

Ir	0.00000000	0.00000000	0.00000000
F	0.00000000	1.23104200	1.39804300
F	1.23104200	0.00000000	-1.39804300
F	0.00000000	-1.23104200	1.39804300
F	-1.23104200	0.00000000	-1.39804300

IrF_4 $^2B_{1g}$ (D_{2h})

Ir	0.00000000	0.00000000	0.00000000
F	0.00000000	1.80716000	0.00000000
F	0.00000000	0.00000000	1.86041500
F	0.00000000	-1.80716000	0.00000000
F	0.00000000	0.00000000	-1.86041500

IrF_4 $^4B_{2g}$ (D_{4h})

Ir	0.00000000	0.00000000	0.00000000
F	0.00000000	1.84418000	0.00000000
F	1.84418000	0.00000000	0.00000000
F	0.00000000	-1.84418000	0.00000000
F	-1.84418000	0.00000000	0.00000000

IrF_4 $^6A_1$ (T_d)

Ir	0.00000000	0.00000000	0.00000000
F	1.11573600	1.11573600	1.11573600
F	-1.11573600	-1.11573600	1.11573600
F	-1.11573600	1.11573600	-1.11573600
F	1.11573600	-1.11573600	-1.11573600

IrF_3 $^1A_1'$ (D_{3h})

Ir	0.00000000	0.00000000	0.00000000
F	0.00000000	1.81795400	0.00000000
F	1.57439400	-0.90897700	0.00000000
F	-1.57439400	-0.90897700	0.00000000

IrF_3 $^3B_1$ (C_{2v})

Ir	0.00000000	0.00000000	0.11738300

F	0.00000000	1.82650300	0.36867300
F	0.00000000	0.00000000	-1.74162400
F	0.00000000	-1.82650300	0.36867300

IrF_3 $^5A_1$ (C_{2v})

Ir	0.00000000	0.00000000	0.12136700
F	0.00000000	1.84407800	0.39689600
F	0.00000000	0.00000000	-1.83215500
F	0.00000000	-1.84407800	0.39689600

IrF_2 $^2A_2$ (C_{2v})

Ir	0.00000000	0.00000000	0.04081900
F	0.00000000	1.78454400	-0.17461400
F	0.00000000	-1.78454400	-0.17461400

IrF_2 $^4\Delta_g$ ($D_{\infty h}$)

Ir	0.00000000	0.00000000	0.00000000
F	0.00000000	0.00000000	1.85380700
F	0.00000000	0.00000000	-1.85380700

IrF $^1\Sigma^+$ ($C_{\infty v}$)

Ir	0.00000000	0.00000000	0.18931600
F	0.00000000	0.00000000	-1.61970800

IrF $^3\Sigma^-$ ($C_{\infty v}$)

Ir	0.00000000	0.00000000	0.19071800
F	0.00000000	0.00000000	-1.63169900

IrF $^3\Phi$ ($C_{\infty v}$)

Ir	0.00000000	0.00000000	0.19506000

F	0.00000000	0.00000000	-1.66884500

Calculated atomic coordinates (in Å) of species for optimized structures at CCSD(T)/aug-cc-pVTZ-PP level.

IrF_5 $^1A_1'$ (D_{3h})

Ir	0.0000000000	-0.0000021894	0.0000000000
F	0.0000000000	1.7858844439	0.0000000000
F	0.0000000000	-0.0000107211	1.9255839565
F	-1.5465875916	-0.8929304062	0.0000000000
F	1.5465875916	-0.8929304062	0.0000000000
F	0.0000000000	-0.0000107211	-1.9255839565

IrF_5 $^1A_1$ (C_{4v})

Ir	0.0000000000	0.0000000000	-0.1136207475
F	0.0000000000	0.0000000000	1.7546770550
F	1.8395213628	0.0000000000	-0.1562561180
F	0.0000000000	-1.8395213628	-0.1562561179
F	-1.8395213628	0.0000000000	-0.1562561180
F	0.0000000000	1.8395213628	-0.1562561179

IrF_5 $^3B_1$ (C_{2v})

Ir	0.0000000000	0.0000000000	-0.0841215721
F	0.0000000000	0.0000000000	1.7418697652
F	-1.8787702202	0.0000000000	-0.1237788603
F	0.0000000000	1.8076245988	-0.3460919093
F	1.8787702202	0.0000000000	-0.1237788603
F	0.0000000000	-1.8076245988	-0.3460919093

IrF_5 $^5B_1$ (C_{4v})

Ir	0.0000000000	0.0000000000	0.0833796243
F	0.0000000000	0.0000000000	-1.8691161046
F	0.0000000000	1.8281909117	0.2747148701

F	-1.8281909117	0.0000000000	0.2747148701
F	0.0000000000	-1.8281909117	0.2747148701
F	1.8281909117	0.0000000000	0.2747148701

IrF_4 $^2B_2$ (D_{2d})

Ir	0.0000000000	0.0000000000	-0.0000000000
F	0.0000000000	1.2270071678	1.3884295643
F	1.2270071678	0.0000000000	-1.3884295643
F	0.0000000000	-1.2270071678	1.3884295643
F	-1.2270071678	0.0000000000	-1.3884295643

IrF_4 $^2B_{3g}$ (D_{2h})

Ir	0.0000000000	0.0000000000	0.0000000000
F	0.0000000000	-1.7987451494	0.0000000000
F	0.0000000000	0.0000000000	1.8508826315
F	0.0000000000	1.7987451494	0.0000000000
F	0.0000000000	0.0000000000	-1.8508826315

IrF_4 $^4B_{2g}$ (D_{4h})

Ir	0.0000000000	0.0000000000	0.0000000000
F	1.8338159512	0.0000000000	0.0000000000
F	0.0000000000	-1.8338159512	0.0000000000
F	-1.8338159512	0.0000000000	0.0000000000
F	0.0000000000	1.8338159512	0.0000000000

IrF_3 $^1A_1'$ (D_{3h})

Ir	0.0000000000	0.0000000000	0.0000000000
F	1.8114360398	0.0000000000	0.0000000000
F	-0.9057180199	-1.5687496278	0.0000000000
F	-0.9057180199	1.5687496278	0.0000000000

IrF_3 $^3B_1$ (C_{2v})

Ir	0.0000000000	0.0000000000	-0.1044083496
F	0.0000000000	1.8198888966	-0.3485354800
F	0.0000000000	0.0000000000	1.7466478094
F	0.0000000000	-1.8198888966	-0.3485354800

IrF_2 $^4\Delta_g$ ($D_{\infty h}$)

Ir	0.0000000000	0.0000000000	0.0000000000
F	0.0000000000	0.0000000000	1.8494035118
F	0.0000000000	0.0000000000	-1.8494035118

IrF_2 $^4\Sigma_g^-$ ($D_{\infty h}$)

Ir	0.0000000000	0.0000000000	0.0000000000
F	0.0000000000	0.0000000000	1.8469905399
F	0.0000000000	0.0000000000	-1.8469905399

IrF_2 $^4\Phi_g$ ($D_{\infty h}$)

Ir	0.0000000000	0.0000000000	0.0000000000
F	0.0000000000	0.0000000000	1.9037004685
F	0.0000000000	0.0000000000	-1.9037004685

IrF $^1\Sigma^+$ ($C_{\infty v}$)

Ir	0.0000000000	0.0000000000	-0.1634695662
F	0.0000000000	0.0000000000	1.6539348080

IrF $^3\Sigma^-$ ($C_{\infty v}$)

Ir	0.0000000000	0.0000000000	-0.1645219864
F	0.0000000000	0.0000000000	1.6645828721

IrF $^3\Phi$ ($C_{\infty v}$)

Ir	0.0000000000	0.0000000000	-0.1673994752
F	0.0000000000	0.0000000000	1.6936964188

IrF $^3\Delta$ ($C_{\infty v}$)

Ir	0.0000000000	0.0000000000	-0.1715145870
F	0.0000000000	0.0000000000	1.7353318550

Calculated atomic coordinates (in Å) of $IrF_4{\cdot}F_2$ complex for optimized structures at B3LYP/aug-cc-pVTZ-PP-D3 level.

$IrF_4{\cdot}F_2$ complex (doublet, side-on)

Ir	0.00000000	0.00000000	0.48672000
F	1.86125900	0.00000000	0.48641700
F	0.00000000	1.80697400	0.48461000
F	0.00000000	-1.80697400	0.48461000
F	-1.86125900	0.00000000	0.48641700
F	0.00000000	-0.69855000	-3.05310700
F	0.00000000	0.69855000	-3.05310700

$IrF_4{\cdot}F_2$ complex (quartet, side-on)

Ir	0.00000000	0.00000000	0.48079400
F	1.84415400	0.00000000	0.48021500
F	0.00000000	1.84424100	0.47955100
F	0.00000000	-1.84424100	0.47955100
F	-1.84415400	0.00000000	0.48021500
F	0.00000000	-0.69879500	-3.01649700
F	0.00000000	0.69879500	-3.01649700

$IrF_4{\cdot}F_2$ complex (doublet, end-on)

Ir	-0.50296600	0.00000000	0.00000000
F	-0.50656800	0.00000100	-1.80617700
F	-0.50494300	-1.86010500	-0.00000100
F	-0.50494500	1.86010500	-0.00000100
F	-0.50656900	0.00000100	1.80617700
F	2.45907600	0.00000000	0.00000000
F	3.86710600	0.00000100	0.00000000

$IrF_4{\cdot}F_2$ complex (quartet, end-on)

Ir	0.00000000	0.00000000	0.52515300
F	0.00000000	1.84345300	0.52810400
F	-1.84345300	0.00000000	0.52810400
F	1.84345300	0.00000000	0.52810400
F	0.00000000	-1.84345300	0.52810400
F	0.00000000	0.00000000	-2.60232400
F	0.00000000	0.00000000	-4.00307000

Calculated atomic coordinates (in Å) for optimized structures at X2C-B3LYP level of theory.

IrF $^3\Phi$ ($C_{\infty v}$):

1c-X2C:

Ir	0.0000000	0.0000000	-0.9306234
F	0.0000000	0.0000000	0.9306234

2c-X2C:

Ir	0.0000000	0.0000000	-0.9309340
F	0.0000000	0.0000000	0.9309340

IrF_2 $^4\Delta_g$ ($D_{\infty h}$)

1c-X2C:

Ir	0.0000000	0.0000000	0.0000000
F	0.0000000	0.0000000	1.8529207
F	0.0000000	0.0000000	-1.8529207

2c-X2C:

Ir	0.0000000	0.0000000	-0.0000003
F	-0.0000000	-0.0000000	1.8595828
F	-0.0000000	-0.0000000	-1.8595825

IrF_3 $^3B_1$ (C_{2v})

1c-X2C:

Ir	-0.0000000	0.0000000	0.3390455
F	-1.8265789	0.0000000	0.5904084
F	1.8265789	0.0000000	0.5904084
F	0.0000000	0.0000000	-1.5198623

2c-X2C:

Ir	0.0000076	-0.0000000	0.3461477
F	-1.8432062	0.0000000	0.5856082
F	1.8432140	0.0000000	0.5856248

F	-0.0000154	0.0000000	-1.5173807

IrF_4 $^4B_{2g}$ (D_{4h})

1c-X2C:

Ir	-0.0000000	0.0000000	0.0000000
F	1.3049003	-1.3049003	-0.0000000
F	1.3049003	1.3049003	0.0000000
F	-1.3049003	-1.3049003	0.0000000
F	-1.3049003	1.3049003	0.0000000

2c-X2C:

Ir	0.0000001	0.0000000	0.0000000
F	1.3073090	-1.3072893	-0.0000000
F	1.3073090	1.3072893	0.0000000
F	-1.3073091	-1.3072890	0.0000000
F	-1.3073090	1.3072890	-0.0000000

IrF_5

1c-X2C: $^3B_1$ (C_{2v})

Ir	0.0000000	0.0000000	-0.0692357
F	-0.0000000	1.8912404	-0.1177466
F	1.8208254	0.0000000	-0.3398580
F	-1.8208254	0.0000000	-0.3398580
F	0.0000000	-1.8912404	-0.1177466
F	-0.0000000	0.0000000	1.7658462

2c-X2C: triplet (C_{4v})

Ir	0.0000002	-0.0000001	-0.0858969
F	1.8586769	-0.0000001	-0.2252280
F	0.0000007	-1.8586862	-0.2252225
F	0.0000006	1.8586860	-0.2252219
F	-1.8586764	-0.0000001	-0.2252259

F	-0.0000020	0.0000005	1.7733872

IrF_6 $^4A_{1g}$ (O_h)

1c-X2C:

Ir	-0.0000000	-0.0000000	0.0000000
F	-1.8491586	-0.0000000	-0.0000000
F	0.0000000	-0.0000000	-1.8491586
F	-0.0000000	1.8491586	0.0000000
F	-0.0000000	-0.0000000	1.8491586
F	0.0000000	-1.8491586	-0.0000000
F	1.8491586	0.0000000	-0.0000000

2c-X2C:

Ir	-0.0000006	0.0000003	-0.0000000
F	-1.8505831	-0.0000005	0.0000001
F	0.0000005	0.0000001	-1.8490661
F	0.0000005	1.8505900	0.0000000
F	0.0000004	0.0000001	1.8490659
F	0.0000006	-1.8505893	0.0000000
F	1.8505817	-0.0000006	0.0000001

IrF_7 $^3A_1'$ (D_{5h})

1c-X2C:

Ir	0.0000000	0.0000000	0.0000000
F	0.0000000	0.0000000	-1.8507642
F	1.8810853	0.0000000	0.0000000
F	0.5812873	-1.7890184	0.0000000
F	0.0000000	0.0000000	1.8507642
F	-1.5218300	-1.1056742	0.0000000
F	-1.5218300	1.1056742	0.0000000
F	0.5812873	1.7890184	0.0000000

2c-X2C:

Ir	-0.0001633	0.0000003	-0.0000001
F	-0.0000806	-0.0000001	-1.8515630
F	1.8830669	-0.0000001	0.0000000
F	0.5819599	-1.7904372	0.0000000
F	-0.0000806	-0.0000001	1.8515629
F	-1.5233310	-1.1068413	0.0000001
F	-1.5233312	1.1068412	0.0000000
F	0.5819600	1.7904374	0.0000001

References

[1] S. Riedel, M. Kaupp, *Angew. Chem. Int. Ed.* **2006**, *45*, 3708–3711.

[2] R. Craciun, D. Picone, R. T. Long, S. Li, D. A. Dixon, K. A. Peterson, K. O. Christe, *Inorg. Chem.* **2010**, *49*, 1056–1070.

[3] W. Jiang, N. J. DeYonker, A. K. Wilson, *J. Chem. Theory Comput.* **2012**, 8, 2, 460–468.
